# How do we define the policy impact of public health research? A systematic review

**DOI:** 10.1186/s12961-017-0247-z

**Published:** 2017-10-02

**Authors:** Kristel Alla, Wayne D. Hall, Harvey A. Whiteford, Brian W. Head, Carla S. Meurk

**Affiliations:** 1 School of Public Health, Faculty of Medicine, The University of Queensland, Herston Road, Herston, QLD 4006 Australia; 2 Queensland Centre for Mental Health Research, The Park Centre for Mental Health, Locked Bag, Archerfield, QLD 4108 Australia; 3 Centre for Youth Substance Abuse Research, The University of Queensland, CYSAR K Floor, Mental Health Centre, Royal Brisbane & Women’s Hospital Campus, Herston, QLD 4029 Australia; 4 School of Political Science, The University of Queensland, St Lucia, QLD 4072 Australia

**Keywords:** Research impact, Policy impact, Evidence-informed policies, Research, Policy, Definitions

## Abstract

**Background:**

In order to understand and measure the policy impact of research we need a definition of research impact that is suited to the task. This article systematically reviewed both peer-reviewed and grey literature for definitions of research impact to develop a definition of research impact that can be used to investigate how public health research influences policy.

**Method:**

Keyword searches of the electronic databases Web of Science, ProQuest, PubMed, EMBASE, CINAHL, Informit, PsycINFO, The Cochrane Database of Systematic Reviews and Google Scholar were conducted between August 2015 and April 2016. Keywords included ‘definition’ and ‘policy’ and ‘research impact’ or ‘research evidence’. The search terms ‘health’, public health’ or ‘mental health’ and ‘knowledge transfer’ or ‘research translation’ were used to focus the search on relevant health discipline approaches. Studies included in the review described processes, theories or frameworks associated with public health, health services or mental health policy.

**Results:**

We identified 108 definitions in 83 publications. The key findings were that literature on research impact is growing, but only 23% of peer-reviewed publications on the topic explicitly defined the term and that the majority (76%) of definitions were derived from research organisations and funding institutions. We identified four main types of definition, namely (1) definitions that conceptualise research impacts in terms of positive changes or effects that evidence can bring about when transferred into policies (example Research Excellence Framework definition), (2) definitions that interpret research impacts as measurable outcomes (Research Councils UK), and (3) bibliometric and (4) use-based definitions. We identified four constructs underpinning these definitions that related to concepts of contribution, change, avenues and levels of impact.

**Conclusion:**

The dominance of bureaucratic definitions, the tendency to discuss but not define the concept of research impact, and the heterogeneity of definitions confirm the need for conceptual clarity in this area. We propose a working definition of research impact that can be used in a range of health policy contexts.

**Electronic supplementary material:**

The online version of this article (doi:10.1186/s12961-017-0247-z) contains supplementary material, which is available to authorized users.

## Background

The measurement of research impact is a contested research and political agenda that poses a complex academic question. Differences in the ways in which evidence might inform policy, and its political underpinnings, highlight key challenges in understanding the policy impacts of research.

The quest to measure and investigate research impact has multiple drivers. Researchers, practitioners and policymakers continue to promote the need for, and benefits of, evidence-informed practice and policies in public health and medicine more generally [1–3]. Government and other funders of research increasingly demand that researchers track the impact of their research to justify research expenditure by showing economic benefits, policy uptake, improved health and community outcomes, industry application and/or positive environmental effects [2, 4–6]. Accountability for research impact is typically embedded in the requirements of grant applications and project reports, in which researchers are required to anticipate the measurable outcomes arising from their proposed research [5, 6]. Within health research, there is an expectation that evidence-based policies and practices may improve the efficiency and effectiveness of health services [2, 6].

Definitions of research impacts are informed (explicitly or implicitly) by what we think knowledge is, what we value about it, and our understanding of the ways in which research evidence can contribute to policy [7]. There is a lack of consensus on how to define research impact [8, 9] and on the terminology used to describe it [10]. Various terms associated with the concept of ‘research impact’, such as knowledge or research ‘translation’, ‘uptake’, ‘diffusion’, ‘utilisation’, ‘payback’, ‘valorisation’, ‘benefits’ and ‘outcomes’, are often used interchangeably [11–13]. Boaz et al. suggest that the “*different terms have a shared interest in change that lies beyond the research process and its primary outputs*” ([13], p. 256), as well as an “*appreciation of the complexity and diversity of research use*” ([13], p. 266). In contrast, other authors note that the lack of standard terminology reflects a deficiency in the literature and call for a clear definition of research impact [14–16].

Many have argued that using a ‘narrow’ approach to measuring research impact results in acknowledging only those types of impact that are easily measured and overlooking those that are hard to measure [2, 17, 18], for example, narrowly defining research impact through the number of citations in the literature (see for example [19]). In contrast, Milat et al. argue that “*The emerging literature on research impact highlights its complex, non-linear, unpredictable nature, and the propensity, to date, to count what can be easily measured, rather than measuring what ‘counts’ in terms of significant, enduring changes*” ([20], p. 2). Greenhalgh and Fahy argue that “*the unenhanced ‘logic model’ of impact, comprising inputs (research funding)→activities (research)→outputs (e.g. papers, guidelines)→outcomes (e.g. changed clinician behaviour, new service models)→impacts (e.g. reduced mortality), is increasingly viewed as over simplistic*” ([18], p. 3). Similarly, Haynes et al. contend that research impact constitutes “*a contested bundle of concepts subject to interpretation and tactical use*” ([21], p. 1047) (see also [22–24]).

This study explores different definitions of research impact, with a specific focus on the applicability of definitions to advancing an academic understanding of how evidence informs health policy. The article presents the findings of a systematic review of the literature to assess how research impact is currently defined in the health literature. On the basis of this review, we propose a working definition of research impact relevant for health policy.

## Methods

This review uses the Preferred Reporting Items for Systematic Reviews and Meta-Analyses (PRISMA) methodological review framework [25] to guide a systematic data collection and critique of the literature defining research impact. The review was conducted to answer three research questions. (1) How is research impact defined in the health literature? (2) What are some of the key constructs underpinning different definitions of research impact? (3) What are some of the implications for research, policy and theory of different ways of defining research impact in the health policy field? The review used mental health research and policy as a case study.

The research questions resulted from discussions among all authors. The search strategies were developed with input from all authors supported by the expertise of a specialist librarian. KA conducted the database searches, assessed the literature against the review criteria, and undertook data extraction, synthesis and analysis of the literature. All authors provided input into findings and conclusions and edited drafts of the article.

### Search strategy

An initial scoping review was undertaken to determine the feasibility of research questions and keywords for the search strategy. The initial focus on mental health policy was expanded to include health policy broadly in the absence of a research impact literature specific to mental health. Heterogeneity of concepts in the research impact literature and the challenge in finding studies that explicitly defined research impact motivated a more expansive search strategy and broader criteria for a definition of ‘research impact’. The peer-reviewed literature was found to be too limited since the majority of definitions were generated within government, not academic, contexts. As Sibbald et al. argue, “*the exclusion of grey literature can skew the results of research syntheses*” ([26], p. 49). The review criteria were expanded to include definitions of impact in all health research found in the grey literature. Grey literature was sourced through (1) Google Scholar, (2) examining reference lists in the included articles, (3) consulting with members of the research team, and (4) contacting external experts.

Electronic databases were searched for peer-reviewed article abstracts and other literature that were evaluated against the review aims and scope. The databases Web of Science, ProQuest, PubMed, EMBASE, CINAHL, Informit, PsycINFO, The Cochrane Database of Systematic Reviews and Google Scholar were searched using a desktop research method tailored to individual databases. Filters were applied to include only articles written in English without time limits. The search of full texts was conducted between August 2015 and April 2016. Keywords included ‘definition’ and ‘policy’ and ‘research impact’ or ‘research evidence’. The search terms ‘mental health’ or ‘public health’ or ‘health’ and ‘knowledge transfer’ or ‘research translation’ were used to focus the search on relevant health discipline approaches. Studies included in the review described processes, theories or frameworks associated with public health, health services or mental health policy.

**Fig. 1:**
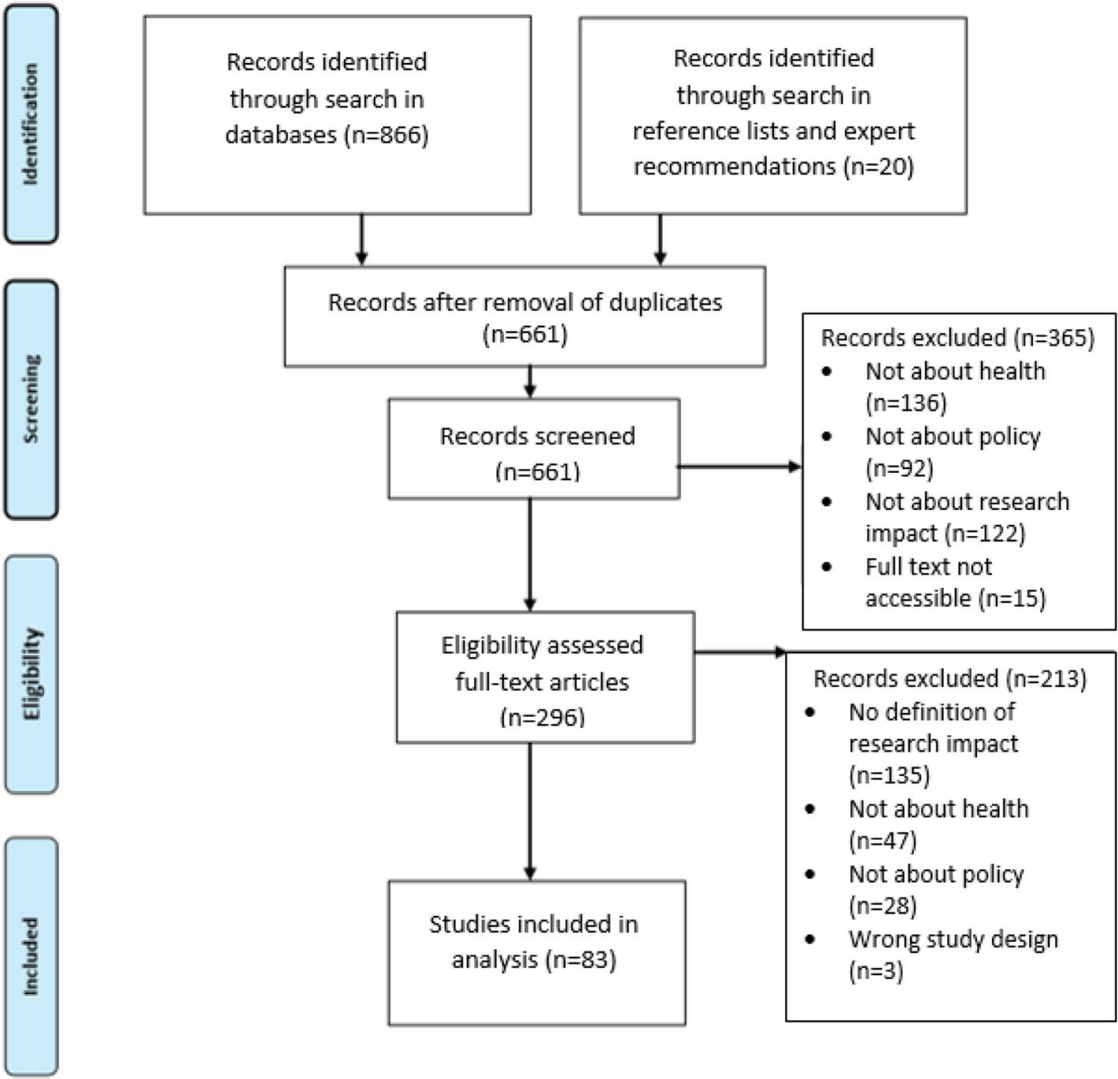
PRISMA flow chart for the systematic review process

Relevant full texts were retrieved and assessed for inclusion against the review criteria. The systematic literature search and review was conducted in the stages depicted in Fig. 1. Reference lists were explored for further relevant resources. Project team members and external experts provided recommendations on the websites of additional key organisations. Data about research impact definitions and constructs were extracted, classified into themes, discussed and synthesised.

### Inclusion criteria

The review imposed no restrictions on the study design apart from excluding unpublished dissertations (n = 3). The sample included theoretical and opinion pieces, case studies, descriptive studies, frameworks and systematic reviews describing processes, methods and conceptual models for assessing research impact. Inclusion criteria were (1) studies addressing public health, mental health, political science or health services disciplines; (2) expressly addressing policy impacts of research as focus or aim; and (3) including an explicit definition of research impact.

A definition of research impact was considered to be present when there was a statement in the article explaining the meaning of research impact (or ‘impact of research’ or ‘policy impact of research’) and there was an explicit effort made to define the term, i.e. an explanatory statement was given such as ‘definition’ or ‘term’ to indicate that research impact ‘is’, ‘denotes’ or ‘is understood as’. In some cases, ‘impact’ rather than ‘research impact’ was defined. When it could be reasonably deduced that the definition referred to research impact, then the definition was included in the study. Texts that described constructs related to research impact but did not define the term(s) were excluded from analysis. All definitions were included from sources that discussed several definitions.

### Data extraction and analysis

Publications were recorded in the reference management software Endnote. Excel spreadsheets were used to record (1) information on the method used in the study; (2) research impact definition(s); (3) source reference(s) of the definition; and (4) constructs extracted from the research impact definitions.

Results were analysed and synthesised in two ways; first, definitions were ordered into types – an ordering that was based largely on the source cited. Second, underlying features of these definitions, based on keywords and constructs evident in definitions, were identified using an inductive comparative method and then categorised into definition types and domains.

## Results

As given in Fig. 1, the search identified 866 sources. Supplemental searches, including reference list searches and expert recommendations, yielded a further 20 publications. After duplicates were removed, 661 titles were screened against the inclusion criteria; 350 articles were excluded during abstract screening because they were not on research impact, health or policy and 135 sources were excluded during full-text screening because they did not contain a definition of research impact. A total of 83 sources were included in this review, including 45 peer-reviewed journal articles, 13 books, 7 conference papers and 18 websites or online reports (Additional file 1). Grey literature comprised 29% of included publications.

### Study characteristics

Only 23% of peer-reviewed journal articles that were on research impact (45 out of 200 that underwent full-text screening) actually defined the term.

The majority of all sources (76%) were published during or after 2011. Half (51%) of the definitions of research impact were from the United Kingdom, 22% from Australia, 16% from other European countries (Germany, Netherlands, Spain, Italy, Austria, Sweden, France and Finland), 10% from the United States of America and 2% from Canada.

A total of 108 definitions were provided. Most publications (60%) referred to a single research impact definition, while the remainder presented two to four definitions. The majority of definitions (76%) were from, or cited, research organisations and funding institutions (i.e. grey literature). The remainder provided original (i.e. unreferenced) definitions of research impact (16%) or cited other peer-reviewed literature (9%). The most highly cited definitions were provided by the United Kingdom Research Excellence Framework (REF), the Higher Education Funding Council for England (HEFCE), the Research Councils UK (RCUK), the Australian Research Quality Framework (RQF) and the Australian Research Council (ARC) (Table 1).

**Table 1:** Total number of definitions referenced (n = 108)

Research organisation or framework referenced	Number of definitions (n)
United Kingdom Research Excellence Framework (REF) and/or the Higher Education Funding Council for England (HEFCE)	30
Research Councils UK (RCUK)	17
Australian Research Quality Framework (RQF) and the Australian Research Council (ARC)	17
London School of Economics and Political Science Public Policy Group (PPG)	5
Economic and Social Research Council (ESRC)	2
Canadian Institute of Health Research (CIHR)	2
Primary Health Care Research and Information Service (PHCRIS)	2
Arts and Humanities Research Council (AHRC)	1
European Science Foundation (ESF)	1
National Health and Medical Research Council (NHMRC)	1
National Institute of Environmental Health Sciences (NIEHS)	1
National Educational Research Forum (NERF)	1
Kellogg Foundation	1
Other definitions referenced	Number of definitions (n)
Original (uncited) definitions (various)	17
Citations to peer reviewed literature (various)	10

Several commonalities were evident in the four main types of definitions identified. These were (1) research impact defined as a demonstrable contribution to society and economy (definition provided by the RCUK); (2) research impact defined as an effect, change or benefit to society and economy (REF and HEFCE); (3) bibliometric definitions; and (4) use-based definitions.

The first two types – the RCUK and the REF/HEFCE – can also be classified as research governance definitions. The research governance group also includes the RQF and ARC definitions, which were hybrids of the RCUK and REF/HEFCE definitions.

### The RCUK definition

The two central definitions used in scientific journals and policy books were contributed by the RCUK and HEFCE (the REF). The RCUK defines research impact as “*the demonstrable contribution that excellent research makes to society and the economy*” [27]. The RCUK defines research impact using the adjective ‘demonstrable’, emphasising that the contribution must be provably linked to an impact (i.e. that the societal impact of research cannot be assumed) whilst the adjective ‘excellent’ equates impact with research quality. In this definition, impact is restricted to the contribution of research to the domains of ‘society’ and ‘economy’. The emphasis on contribution (input) makes this definition neutral with respect to having an expectation of a specific outcome or change.

While the RCUK does not explicitly reference policy impacts, others have expanded upon it to encompass research impacts on policy in two slightly different ways; first, is the impact of research to an area policy (as in [28]), i.e. the ‘policy benefits’ of research [29] and second is the contribution that research can make to policy and good governance, i.e. to improving the effectiveness of public services and policy [30, 31].

### The REF/HEFCE definition

The HEFCE manages the REF, which is used to assess research quality and allocate research funding in the United Kingdom [32]. HEFCE and the REF research impact definitions are equivalent and referenced interchangeably.

The HEFCE/REF guidelines define research impact as “*an effect on, change or benefit to the economy, society, culture, public policy or services, health, the environment or quality of life, beyond academia*” [32]. These two core definitions – research impact as a ‘demonstrable contribution’ (by RCUK) versus ‘effect on, change or benefit’ to society, policy and economy – differ in the inclusivity of their concept of impact, on whether the process (contribution) or outcome (effect) is emphasised, and whether research impact can be readily measured.

The HEFCE/REF is conceptually more nuanced than that provided by the RCUK insofar as it emphasises a broader range of areas of influence. Chandler [33] adds to the core definition that research impact enables the development of new products, services and policies – in other words, research impact can be defined through its capacity to facilitate innovation. Similarly, Donovan [34] adds industry and government to the list of ‘beneficiaries’ of research impact. Pragmatic and person-centred interpretations of research impact require that research impact translates into ‘real-world outcomes’ [35] and some, such as Chandler [33], see research impact as pertaining to economic, social and cultural ‘lives’, and thus referencing (individual) human activities.

Multiple authors cite the second part of the REF/HEFCE definition, which includes a list of impact foci, namely “*activity, attitude, awareness, behaviour, capacity, opportunity, performance, policy, practice, process or understanding of an audience, beneficiary, community, constituency, organisation or individuals in any geographic location whether locally, regionally, nationally or internationally"* ([36], p. 5; [37], p. 45). This definition broadens the spheres of possible impact to include psychosocial impacts and impacts at numerous organisational and geographical scales.

REF departs substantially from the RCUK definition insofar as it includes within the definition the role of research in the prevention of harms and reducing risks, costs or negative impacts [18]. The normative rendering of research impact as a benefit or a positive return (rather than the value-neutral ‘change’ and ‘effect’) is the focus of Ovseiko et al.’s [38] definition. This extends the HEFCE definition to include ‘social value’ and specify positive returns from research in terms of social cohesion, social welfare and investments, public engagement with science, and sustainable development. Reed [12] specifies that research evidence can be useful in preventing the adoption of harmful legislation and products.

### The ARC and the Australian RQF definitions

The third most frequently cited definition, that of the research funding body the ARC, is a hybrid of the REF and RCUK definitions and so cannot be classified as a distinct type. This is the broadest core definition included within this review with respect to the areas of potential impact that the definition encompasses. The ARC [39] defines research impact as “*demonstrable contribution that research makes to the economy, society, culture, national security, public policy or services, health, the environment, or quality of life, beyond contributions to academia*” (for example [40], p. 158; [41], p. 32). National security is a unique feature of the ARC cited definitions. The ARC definition includes policy impacts of research within its core definition and regards a wide range of different types and levels of impact that are left open for further inclusion.

The common unifying elements between the ARC [39] and the Australian RQF [42] definitions are (1) a reference to the social, economic, cultural and environmental benefits of research and (2) extension of impact scope beyond academia. Some definitions phrased these four contributions as ‘outcomes’ instead of benefits [14, 34]. They differed insofar as publications citing the RQF definition of research impact were more heterogeneous, narrative and interpretative than those referring to the ARC. Policy impacts were explicitly mentioned in all five publications that used the ARC definition. However, only one source that cited the RQF mentioned policy impacts.

### Bibliometric definitions

Bibliometric definitions, some of which arise out of the field of economics, focus on demonstrable and measurable research impacts in the form of quantifiable data. Some authors, such as Tonta et al. [19], approach research impacts quite narrowly and define research impact quantitatively as citation frequency in literature. However, others are more inclusive and list other forms of quantifiable impacts as part of their definition. Research impact for the London School of Economics and Political Science Public Policy Group (PPG) is a “*recorded or otherwise auditable occasion of influence from academic research on another actor or organization*” ([43], p. 310; [44], p. 7). The PPG website [45] adds to this definition by indicating that “*impact is usually demonstrated by pointing to a record of the active consultation, consideration, citation, discussion, referencing or use of a piece of research*”. This approach considers research impact assessment methods beyond citations by attempting to capture oral communication, but it demands a record of impacts. The Association of Commonwealth Universities [46] cites the PPG definition and states that research impact establishes the influence of research knowledge, rather than its consequences.

Similarly, Hannemann-Weber et al. [47] explain research impact through quantifiable influence and draw direct links between activities and research outputs referred to as ‘impact of publications’. While focussed on bibliometrics, this conceptualisation acknowledges broader social processes that underpin research impact as measured bibliometrically, such as the acceptability and visibility of research, the status (reputation) of research producers and the actions of researchers in the promotion of research findings. The explanation of research impact through quality, visibility and reputation of research outputs thus provides a definition that interestingly overlaps with the RCUK’s alignment of research impact with research quality. Moed et al. ([48], p. 132) formulate a definition in which they clarified the relationship between research outputs (‘the extent to which the research creates a body of scientific results’) and impact (‘the actual influence of the research output on surrounding research activities’).

Qin [49] agrees with these ideas in defining research impact by the extent to which outputs are diffused across disciplinary and geographical boundaries (measured by citations), the extent to which these have been adopted (measured by intellectual property purchases and licences), and benefits established (measured quantitatively and qualitatively). Harland [50], citing Korhonen et al. [51], expands on the list of research outputs that constitute evidence of impact by adding the concept of ‘pathways’, notably international and cross-national platforms, that can improve impact, albeit still defining research impact narrowly, in terms of dissemination in academic circles. Nightingale and Marshall [52] expressed the idea that citations exhibit the extent of academic significance, noting, however that this is not the same thing as research impact.

The Australian National Health and Medical Research Council [53] defines citation tracking as one expression of research impact in terms of the impact of ideas and methods within academia. However, the National Health and Medical Research Council definition acknowledges that there are also less easily measurable forms of research impact such as research that improves patient care, guides policymakers to adopt health prevention strategies or translates into systems level change. Hartwell et al. [54] suggested that only research that affects practice has impact regardless of how highly cited it is. Cohen et al. [55] agree that policy impacts of research have broad effects, and can result from pro-health campaigns and from organisational and funding changes. For Cohen et al. [55] policy impacts must be tangible, measurable and manifest in a specific time frame, namely after research had been produced without feeding back into research production.

### Use-based definitions

Many academic articles define research impact by distinguishing between research impact, research use and research outputs. Unlike the instrumentalist definitions found in the grey literature, these definitions tend to be more theoretical, policy and practice oriented, and focussed on the influence of research findings on the activities and knowledge of researchers and policymakers.

Walter et al. [56] defined research impacts in terms of the uses to which it is put, namely, conceptual use versus instrumental use. An extended form of this definition is provided by Nutley et al.:

“*Broadly, instrumental use refers to the direct impact of research on policy and practice decisions. It identifies the influence of a specific piece of research in making a specific decision or in defining the solution to a specific problem, and represents a widely held view of what research use means. Conceptual use is a much more wide-ranging definition of research use, comprising the complex and often indirect ways in which research can have an impact on the knowledge, understanding and attitudes of policy makers and practitioners. It happens where research changes ways of thinking, alerting policy makers and practitioners to an issue or playing a more general ‘consciousness-raising’ role*” ([24], p. 36).

Meagher et al. [57] emphasise that instrumental research impact deals with the attribution of particular policy decisions to specific research whereas conceptual impact embodies the significance of diffusion of research impacts. Their definition is different to the outcomes/benefits-based definitions. Instrumental use is understood in terms of the metaphor of a ‘hammer’. Research ‘hits’ policy and practice to cause a decision or directive. Impact, here, is causal but not necessarily linked to outcomes (beneficial or otherwise).

Jones and Cleere [30] reference the European Science Foundation in defining research impacts in terms of both their contributions to specific fields and in terms of how they are enacted. This included health impacts (‘contribution to public health, life expectancy, prevention of illnesses and quality of life’) and policy impacts of research (‘contribution to how policymakers act and how policies are constructed and to political stability’). Thus, research impacts on policy can be manifested through contributions to the political culture, the policy development process and the stability of the political regime.

Brewer [8] argues that policy-specific impacts are demonstrated in research use by policymakers, research uptake into policies, and by improved effectiveness of policies and health services. Wilkinson et al. [58] also stress that the policy impacts of research extend to private and non-governmental sectors. Their broad definition encompasses the processes of knowledge exchange and relationships that facilitate research impact.

### Comparison of domains found in definitions

Definitions each varied on one of four domains of meaning, namely contribution, change, avenues and levels of impacts (Table 2).

**Table 2:** Domains of research impact definitions

Domain	Summary of domain, including keywords and count across definitions^a^ (n = 108)	Count across constructs in domain (n)
Contribution	Specific areas of focus, including economy (72), society (58), policies (56), environment (52), culture (51), health (49), quality of life (43), services (42), community (24), organisations (24), practices (18), security (9)	465
Avenues	The different elements and processes by which research can have impact, including academic (scholarly) (36) activities (24), knowledge (22), understanding (19), processes (17), excellent research (16), attitudes (13), awareness (10), funding (6), ideas (4)	184
Change	Synonyms of impact evident in definitions, including benefit (58), change (39), effect (36), contribution (29), negative (consequences) or harm (6), positive returns (6), (reduction of) risk (2)	138
Levels	Scale or sphere of impact that research evidence can have, including individual (20), national (19), international (14), local (10), global (9), regional (9)	81

^a^Where keywords were repeated in a definition, they were only counted once

Research impact was most often defined in terms of the contribution that research made to different areas of influence, including among others the economy, society, environment, culture, policies and health. Just over half (52%) of definitions explicitly mentioned policy as an object of research impact.

Research impact definitions also varied concerning the types of avenues of impact, i.e. the mechanisms or processes by which research could be said to have impact. This was the second predominant construct found in definitions. Effects on knowledge, understanding, awareness and/or attitudes (for example, of practitioners and policymakers) were included in 59% of research impact definitions. References to ‘activities’ (22% of definitions) and ‘processes’ (16%) were also frequent. One-third (33%) of definitions distinguished research impacts as being those evident beyond academia. Many publications defined impact in terms of ‘outcomes’ achieved (14%) and ‘outputs’ (13%). Research impact was defined in terms of ‘demonstrable’ or ‘measurable’ outcomes in 28% of definitions. Two main aspects emerged from definitions, namely (1) research has impact by changing knowledge, understanding, awareness and attitudes, or through creating products (effects on different avenues of impact); and (2) research has impact through scholarly activities or excellent research (through effects on quality and behaviour).

Another important element was the variety of synonyms for impact that existed, i.e. as an effect, change or benefit to areas of influence including any positive and negative impacts that research may have. A clear ‘positivity bias’ was evident in these definitions, indicating their origins in bureaucratic documents. Importantly, over half (58%) of all definitions interpreted research impact as leading to positive gains or the reduction in societal harms; no definitions mentioned that research use may also lead to negative outcomes.

Finally, the research impact construct was also commonly defined through a range of levels of impacts that research evidence can have (i.e. international, national, local and individual impacts). References to the individual and national levels of research impact received most attention and were respectively mentioned in 19% and 18% of research impact definitions. Global and regional research impacts were mentioned in less than 10% of definitions.

## Discussion

### The evolution, diffusion and use of research impact definitions

This review confirmed the heterogeneous and recombinant nature of research impact definitions indicating, perhaps, struggles to find an acceptable definition for this complex term [8, 9, 59]. However, our review also highlighted that most of the research impact literature discusses this concept without explicitly defining it, with only one-fifth of peer-reviewed journal articles doing so. Attempts to define research impact were more common in the grey than in the peer-reviewed literature, confirming the extent to which impact is a bureaucratic rather than academic term [60, 61]. Our findings confirm previous research showing that research impact definitions, and the research impact ‘agenda’, have emanated from research funding bodies in the United Kingdom and been diffused to other countries, such as Australia [60, 62].

The dominance of research governance definitions indicated by our review, reflects the political history of the impact agenda [62, 63]. However, their continued dominance may limit the pursuit of academic understandings of research impact by restricting it to demonstrable returns from research investments. Our review found evidence of this type of focus in nearly a third of research impact definitions. While some authors, like Kenyon [15], express scepticism as to whether it is in fact possible to define such a complex term in an adequate manner, we echo the views of authors such as Tinkler [64] and Bornmann [63], who advocate for the need to include ‘the diversity and richness’ of research impacts into definitions; we argue that this entails the need for researchers to exercise reflexivity in relation to research impact definitions, being mindful of the origins of the different definitions, their purposes and limitations.

### Debates over definitions

Most definitions of research impact emphasised positive returns. While some definitions used more neutral language, negative impacts of research were rarely mentioned. The issue from a research governance standpoint is whether a focus on impacts defined in terms of non-academic benefits creates an incentive to skew results to demonstrate benefit, even where there is none. This may create perverse incentives to implement ideas before they have been properly tested or their implications fully thought through. Most definitions interpreted research impact as leading to positive gains or the reduction in societal harms. However, there are several examples of research that has had negative or, at least, contested impacts (e.g. drugs such as thalidomide or weapons of mass destruction). Researchers may be encouraged to conduct research in favour of short-term ‘impacts’ with the result that research that is critical of prevailing governing paradigms is not pursued and not funded, resulting in longer term negative effects on innovation and advancement through research. Furthermore, a definition that encompasses a clear ‘positivity bias’, as is evident in these definitions, may be limited in pursuing academic understanding of how evidence impacts policy.

A related concept to that of ‘research impact’ is that of ‘knowledge valorisation’. Knowledge valorisation is gaining significant traction in the European Union research funding and dissemination discourse. Valorisation is a process by which academic knowledge is transformed into social and economic value [65, 66]. Valorisation focusses on the process of value creation from academic research through commercial activities and industry associations with academia; in other words, it is closely associated with the commercialisation of academic research [67].

Valorisation is a concept that is linked to, but not the same as, a definition of research impact. Valorisation and impact are linked through their combined focus on the usefulness of research, and the ability to produce commercial and/or social returns from academic knowledge. Perhaps due to its focus on commercialisation, the literature on valorisation has paid less attention to policy impacts of research. Furthermore, Benneworth [67] has critiqued the conceptualisation of knowledge valorisation for being more applicable to the physical and life sciences than to the humanities and social sciences. In contrast, ‘research impact’ definitions provide a broader and more abstract conceptualisation concerned with the longer term application of knowledge to more complex societal problems.

Conceptualisations of ‘knowledge valorisation’ and ‘research impact’ both face the same issue in terms of some lack of conceptual clarity and approach [66, 68].

### How policy features in research impact definitions

Around half of the research impact definitions included a consideration of how research impacts on policy, mostly by mentioning policy as one of several impact foci. The complexities involved in the conceptualisation of research impact on policy have been acknowledged by many authors [7, 10, 13]. There is a recursive issue here, among the main challenges of defining research impact on policy are the uncertainties regarding how exactly research evidence brings about policy changes, and also how those policy changes link to ‘real-world’ outcomes [55, 63]. However, these uncertainties exist, in part, due to a lack of agreed upon definitions of research impact that can facilitate a research agenda.

There are several recognised difficulties in attributing a policy impact to a specific piece of research [5, 69, 70]. The original piece of research may be re-interpreted in the policy process in ways that are incorrect or not consistent with its intent, or it may be adapted to particular contexts and transformed in the process. Multiple influences at different stages of research and policy translation may also function to diffuse knowledge. Policy change, as suggested by Thomas [71], is dynamic and the product of a web of decisions that may reflect competing values that result in political compromises. A policy relevant definition of research impact should take account of the fact that there is not always a direct pathway from evidence to policy and that impact can be more, or less, directly identifiable – depending on whether that impact is conceptual or instrumental [22, 24].

Consequently, for a research impact definition to adequately capture the complexities of policy impacts, it must include elements that relate to two different phenomena – policy content and policy processes. We need a definition that is clear about the different, both direct and indirect, ways in which research can impact on policy and thus help us investigate it for academic purposes.

### Proposed definition of research impact for (mental) health policy

Based on this review, we propose the following definition for research impact on health policy that can be tailored for use in health disciplines, including public and mental health. A definition specific to mental health is given:

*Research impact is a direct or indirect contribution of research processes or outputs that have informed (or resulted in) the development of new (mental) health policy/practices, or revisions of existing (mental) health policy/practices, at various levels of governance (international, national, state, local, organisational, health unit).*

This definition tailors core constructs that were identified in the literature to the field of health policy. It includes the constructs of contribution (but not demonstrable), change, research outputs, policies, practices, various avenues and levels of impacts and encompasses impacts that may be said to occur at different time points. For example, immediate impact might be the use of research processes and outcomes to increase policymakers’ knowledge and inform attitudes, medium-term impact may be an impact of research on the development and revision of policy, and a long-term outcome may be the multilevel impact of research through the implementation and evaluation of policy and practice.

The definition overcomes some of the limitations of existing definitions. It does not restrict research impact to its measurable qualities and includes both desirable and undesirable impacts, allowing for its use in different contexts to capture the full range of possible research impacts. Of the definitions available, the proposed definition is perhaps most similar to that of the ARC [39] definition.

### Strengths, limitations and future research

The key strength of this review is its comprehensiveness and wide coverage of both peer-reviewed and grey literature, the latter having been neglected in previous reviews. The use of a systematic search methodology allowed us to identify the prevalence and reach of different types of definition and research impact definitions overall. The review confirmed that the two most common definitions in both peer-reviewed and grey literature originated from the grey literature, supporting the need to include the grey literature in future reviews of research impact studies.

This review is limited by its conservative search term selection, as only publications that explicitly used the term ‘research impact’ or its close derivatives were included. It is possible that relevant literature that failed to use this terminology was excluded, for example, economic literature on payback models [72, 73]. That said, economic models such as the payback model arguably represent operational definitions rather than conceptual definitions.

The study focus is limited to literature that was in English. Thus, it may not have captured relevant discourses from European or non-English speaking literature.

Additionally, five unique definitions were identified in this review that fell outside the typologies constructed [61, 74–77]. These definitions all drew distinctions between the ideas of impacts, outputs and/or outcomes, and shared some of the features of the aforementioned definitions. Future research will take account of feedback from relevant stakeholders (e.g. researchers and policymakers) on different ways of defining research impact and on the definition proposed here; academics, policymakers, bureaucrats, clinicians, patients and the general community are likely to hold different views on this topic.

## Conclusion

Facilitating the effective translation of health research to policy and practice requires a dedicated research agenda. The dominance of bureaucratic definitions, the tendency to discuss but not define the concept of research impact, and the heterogeneity of definitions confirm the need for conceptual clarity in this area. Without wanting to impose a reductive imperative within debates around research impact definitions, we pose a definition of research impact that is primarily for the purposes of academic study of the impact of research on health policy but that could be adapted for use in other specific contexts.

## Additional file

Additional file 1: Definitions of research impact included in the study. **Table S1**. RCUK definitions. **Table S2**. REF/HEFCE definitions. **Table S2a**. Research impacts as benefits, effects or changes (REF/HEFCE not cited). **Table S3**. ARC/RQF definitions. **Table S4**. Bibliometric definitions. **Table S5**. Use-based definitions. **Table S6**. Original definitions. (DOCX 58 kb)

## Abbreviations

ARC: Australian Research Council
HEFCE: Higher Education Funding Council for England
PPG: London School of Economics and Political Science Public Policy Group
RCUK: Research Councils UK
REF: United Kingdom Research Excellence Framework
RQF: Australian Research Quality Framework
