# Supplementary material for: How do we define the policy impact of public health research? A systematic review
Source: Health Res Policy Syst. 2017 Oct 2;15:84. doi: 10.1186/s12961-017-0247-z (PMC5625646; doi:10.1186/s12961-017-0247-z)
Supplement: Additional file 1: — Definitions of research impact included in the study. Table S1. RCUK definitions. Table S2. REF/HEFCE definitions. Table S2a. Research impacts as benefits, effects or changes (REF/HEFCE not cited). Table S3. ARC/RQF definitions. Table S4. Bibliometric definitions. Table S5. Use-based definitions. Table S6. Original definitions. (DOCX 58 kb) [file 12961_2017_247_MOESM1_ESM.docx]

# **Article titled ‘How do we define the policy impact of public health research? A systematic review’**

# **Additional file 1. Definitions of research impact included in the study**

# **Tables 1-6: Types of research impact definitions**

# **Table 1. RCUK definitions**

| *Author of Reference* | *Reference Type* | *Definition* | *Reference to Source of Definition* |
| --- | --- | --- | --- |
| 1. **Bannister and Hardill [1]** | Journal article | “The Research Councils of the UK (RCUK), responsible for the expenditure of about three billion pounds a year public resource on academic research, have also moved to prioritise impact though RCUK adopts a broader definition of impact as ‘the demonstrable contribution that excellent research makes to society and the economy’, embracing ‘all the diverse ways that research-related skills benefit individuals, organisations and nations.” (p. 1) | Research Councils UK (RCUK) |
| 1. **Brewer [2]** | Book | “Research Councils United Kingdom (RCUK), the umbrella body for all the research councils, defines research impact as ‘the demonstrable contribution that excellent research makes to society and the economy.” (p. 89) | Research Councils UK (RCUK) |
| 1. **Chandler [3]** | Book | “…the Research Councils UK (RCUK) emphasise the link with excellence in their definition: ‘Impact is the demonstrable contribution that excellent research makes to society and the economy…’” (p. 2) | Research Councils UK (RCUK) |
| 1. **Clegg, Lightfoot and Scully [4]** | Conference paper | “The Research Councils UK (RCUK) definition of research impact is 'the demonstrable contribution that excellent research makes to society and the economy’.” (p. 222) | Research Councils UK (RCUK) |
| 1. **Economic and Social Research Council [5]** | Website | “Research Councils UK (RCUK) defines research impact as 'the demonstrable contribution that excellent research makes to society and the economy’.” | Research Councils UK (RCUK) |
| 1. **Halse and Mowbray [6]** | Journal article | “Research Councils UK, for example, has defined impact as ‘recognising the diverse ways in which research can contribute to the UK economy, including social, environmental, cultural, health and policy benefits as well as more obvious economic benefits’.” (p. 515) | Research Councils UK (RCUK) |
| 1. **Holt and Lewis [7]** | Book | “Research Councils UK (RCUK) invests around £3 billion each year in university research, including psychological research. They define research impact as ‘the demonstrable contribution that excellent research makes to society and the economy’ which is ‘of benefit to `individuals, organisations and nations’.” (p. 210) | Research Councils UK (RCUK) |
| 1. **Jansson, Karvonen, Kettunen and Ollus [8]** | Web report | “Considering the research perspective, one can consider the definition of research impact by the Research Councils UK (RCUK), which is ‘the demonstrable contribution that excellent research makes to society and the economy’. They go on to state that it contains all the diverse ways that research-related skills benefit individuals, organisations and nations.” (p. 2) | Research Councils UK (RCUK) |
| 1. **Jones and Cleere [9]** | Web report | “The Research Councils UK makes an important distinction between academic impact and socio-economic impact in the following ways: ‘Academic impact: The demonstrable contribution that excellent research makes to academic advances, across and within disciplines, including significant advances in understanding, methods, theory and application.’ ‘Economic and societal impacts: The demonstrable contribution that excellent research makes to society and the economy. Economic and societal impacts embrace all the extremely diverse ways in which research-related knowledge and skills benefit individuals, organisations and nations by: fostering global economic performance, and specifically the economic competitiveness of the United Kingdom, increasing the effectiveness of public services and policy, enhancing quality of life, health and creative output’.” (p. 17) | Research Councils UK (RCUK) |
| 1. **Kraatz, Hampson, Parker and Roos [10]** | Book | ‘In the UK, the Research Councils UK (2012) has adopted a very broad definition of research impact to include benefits across many diverse economic, social and environmental areas of concern including quality of life. In terms of impacts on the economy, measures relating to economic growth, productivity and reduced costs have introduced potential metrics into the process.’ (p. 304) | Research Councils UK (RCUK) |
| 1. **Marcella, Lockerbie and Cameron [11]** | Conference paper | “Research Councils UK defines impact as ‘the demonstrable contribution that excellent research makes to society and the economy’.” (p. 2) | Research Councils UK (RCUK) |
| 1. **Reed [12]** | Book | “More simply, Research Councils UK defines research impact as ‘the demonstrable contribution that excellent research makes to society and the economy’.” (p. 181) | Research Councils UK (RCUK) |
| 1. **Research Councils UK (RCUK) [13]** | Website | ‘RCUK defines impact as the demonstrable contribution that excellent research makes to society and the economy. This occurs in many ways – through creating and sharing new knowledge and innovation; inventing ground breaking new products, companies and jobs; developing new and improving existing public services and policy; enhancing quality of life and health; and many more.’ | No reference (original) |
| 1. **Shaw [14]** | Book | “The UK funding councils define impact as ‘the demonstrable contribution that excellent research makes to society and the economy.’ Key aspects of this definition of research impact are that impact must be demonstrable and that one cannot have impact without excellence. The research councils express it as follows: ‘We aim to achieve research impact across all our activities. This can involve academic impact, economic and societal impact or both: Academic impact is the demonstrable contribution that excellent social and economic research makes to scientific advances, across and within disciplines, including significant advances in in understanding, method, theory and application. Economic and societal impact is the demonstrable contribution that excellent social and economic research makes to society and the economy, of benefit to individuals, organisations and nations.’” (p. 259) | Research Councils UK (RCUK) |
| 1. **Shaw and Holland [15]** | Book | “The latter [research councils] define research impact as ‘the demonstrable contribution that excellent research makes to society and the economy. /-/ Academic impact is the demonstrable contribution that excellent social and economic research makes to scientific advances, across and within disciplines, including significant advances in in understanding, method, theory and application. Economic and societal impact is the demonstrable contribution that excellent social and economic research makes to society and the economy, of benefit to individuals, organisations and nations.” (p. 6855-6867) | Research Councils UK (RCUK) |
| 1. **SOAS University of London [16]** | Website | “Impact is defined as follows by RCUK: ‘…. the demonstrable contribution that excellent research makes to society and the economy. Research impact embraces all the diverse ways that research-related skills benefit individuals, organisations and nations. These include: fostering global economic performance, and specifically the economic competitiveness of the United Kingdom increasing the effectiveness of public services and policy, enhancing quality of life, health and creative output’.” | Research Councils UK (RCUK) |
| 1. **Weitkamp [17]** | Journal article | “For example, the UK Research Councils (RCUK) defines research impact as ‘the demonstrable contribution that excellent research makes to society and the economy’. The RCUK go on to explain that these impacts might occur in areas as diverse as the economy, policy and health and wellbeing.” (p. 1.) | Research Councils UK (RCUK) |

# **Table 2. REF/HEFCE definitions**

| *Author of Reference* | *Reference Type* | *Definition* | *Reference to Source of Definition* |
| --- | --- | --- | --- |
| 1. **Association of Commonwealth Universities (ACU) [18]** | Website | “The UK’s Research Excellence Framework (REF) defines impact as ‘reach’ and ‘significance’ and can encompass the ‘effect on, change or benefit to the economy, society, culture, public policy or services, health, the environment or quality of life, beyond academia’.” | UK Research Excellence Framework (REF) |
| 1. **Bannister and Hardill [1]** | Journal article | “In the UK, the Research Excellence Framework (REF), the tool intended to judge the quality of academic performance, includes impact as a key component of its intended evaluation. The REF defines impact as ‘an effect on, change or benefit to the economy, society, culture, public policy or services, health, the environment or quality of life beyond academia’.” (p. 168) | Higher Education Funding Council for England (HEFCE) |
| 1. **Bernal [19]** | Journal article | "According to the UK Research Evaluation Framework (REF), impact means ‘any effect on, change or benefit to the economy, society culture, public policy or services, health, the environment or quality of life, beyond academia’, thus going beyond the traditional interpretation of impact as scholarly excellence." (p.71) | UK Research Excellence Framework (REF) |
| 1. **Brewer [2]** | Book | “The definition pursued in the REF is different in crucial respects. HEFCE’s website says the following about impact. ‘For the purposes of the REF, impact is defined as an effect on, change or benefit to the economy, society, culture public policy or services, health, the environment or quality of life, beyond academia. Impact includes, but is not limited to, an effect on, change or benefit to: the activity, attitude, awareness behaviour, capacity, opportunity, performance, policy, practice, process or understanding of an audience, beneficiary, community, constituency, organisation or individuals in any geographic location...’” (p. 90) | UK Research Excellence Framework (REF) |
| 1. **Chandler [3]** | Book | “…and the Research Excellence Framework ‘REF2014’ defines impact as ‘… an effect on, change or benefit to the economy, society, culture, public policy or services, health, the environment or quality of life, beyond academia’.” (p. 2) | UK Research Excellence Framework (REF) |
| 1. **Harris [20]** | Book | “For example, the UK government’s 2014 Research Excellence Framework will for the first time explicitly assess the impact of research beyond academia. The framework defines impact as ‘any effect on, change or benefit to the economy, society, culture, public policy or services, health, the environment or quality of life, beyond academia’.” (p. 26) | UK Research Excellence Framework (REF) |
| 1. **Colley [21]** | Journal article | “‘Impact’ is defined by the REF guidelines as ‘an effect on, change or benefit to the economy, society, culture, public policy or services, health, the environment or quality of life, beyond academia’ generated by excellent research.” (p. 1) | UK Research Excellence Framework (REF) |
| 1. **Détourbe [22]** | Journal article | “In the REF, impact was exclusively understood as impact outside the academic field and was defined broadly as ‘effect on, change or benefit to the economy, society, culture, public policy or services, health, the environment or quality of life, beyond academia.’/-/ Impact includes, but is not limited to, an effect on, change or benefit to: the activity, attitude, awareness, behavior, capacity, opportunity, performance, policy, practice, process or understanding; of an audience, beneficiary, community, constituency, organization or individuals; in any geographic location whether locally, regionally, nationally or internationally’.” (p. 9) | UK Research Excellence Framework (REF) |
| 1. **Doyle, Farley, Keppell, Cuthill and McDonald [23]** | Conference paper | “HEFCE defines research impact as ‘an effect on, change or benefit to the economy,  society, culture, public policy or services, health, the environment or quality of life, beyond academia’.” (p. 639) | Higher Education Funding Council for England (HEFCE) |
| 1. **Doyle and McDonald [24]** | Website | "The REF is administered by the Higher Education Funding Council for England (HEFCE) that defines research impact as ‘an effect on, change or benefit to the economy, society, culture, public policy or services, health, the environment or quality of life, beyond academia’." (p. 3) | UK Research Excellence Framework (REF) |
| 1. **Greenhalgh and Fahy [25]** | Journal article | “In the 2014 REF, 20 % of the score (and hence funding allocation) for each higher education institution was awarded for impact [1]. Impact was defined as occurring when academic research led to ‘… benefits to one or more areas of the economy, society, culture, public policy and services, health, production, environment, international development or quality of life, whether locally, regionally, nationally or internationally’ (paragraph 62) and as ‘…manifested in a wide variety of ways including, but not limited to: the many types of beneficiary (individuals, organisations, communities, regions and other entities); impacts on products, processes, behaviours, policies, practices; and avoidance of harm or the waste of resources’.” (p. 2) | UK Research Excellence Framework (REF) |
| 1. **Halse and Mowbray [6]** | Journal article | ‘Similarly HEFCE and Star Metrics have operationalised research impact as the benefits and returns that research brings to the economy, environment, international and social arenas, public policy and services, and to the quality of life, culture and health of individuals and communities.’ (p. 515) | Higher Education Funding Council for England (HEFCE) and Star Metrics |
| 1. **Hartwell, van Teijlingen and Parker [26]** | Journal article | “The REF defines impact as ‘any identifiable benefit to or positive influence on the economy, society, public policy or services, culture, the environment and/or quality of life’. Impact includes, but is not limited to, an effect on, change or benefit to: the activity, attitude, awareness, behaviour, capacity, opportunity, performance, policy, practice, process or understanding; of an audience, beneficiary, community, constituency, organisation or individuals; and in any geographic location whether locally, regionally, nationally or internationally./-/ The following cannot be claimed as impact under the REF definition: impact on teaching undergraduate or postgraduate students; anticipated impact (i.e. no evidence yet); or dissemination to the public (without evidence of impact or benefit from that activity). This definition excludes dissemination activities and academic impact, so, for example, an excellent academic study on consumer theory which is widely quoted by fellow researchers but does not seem to affect practice, would not count as having impact.” (p. 75-76) | Higher Education Funding Council for England (HEFCE) |
| 1. **Higher Education Funding Council for England (HEFCE) [27]** | Website | “The Research Excellence Framework was the first exercise to assess the impact of research outside of academia. Impact was defined as ‘an effect on, change or benefit to the economy, society, culture, public policy or services, health, the environment or quality of life, beyond academia’.” | UK Research Excellence Framework (REF) |
| 1. **Holt, Goulding and Akintoye [28]** | Journal article | “[…] impact is defined as an effect on, change or benefit to the economy, society, culture, public policy or services, health, the environment or quality of life, beyond academia.” (p. 20) | Higher Education Funding Council for England (HEFCE) |
| 1. **Jones and Cleere [9]** | Web report | “Meanwhile in the UK, the Research Excellence Framework (REF) defines impact as:  ‘an effect on, change or benefit to the economy, society, culture, public policy or services, health, the environment or quality of life, beyond academia. Impact includes, but is not limited to, an effect on, change or benefit to: the activity, attitude, awareness, behaviour capacity, opportunity, performance, policy, practice, process or understanding of an audience, beneficiary, community, constituency, organisation or individuals in any geographic location whether locally, regionally, nationally or internationally. Impact includes the reduction or prevention of harm, risk, cost or other negative effects’.” (p. 17) | UK Research Excellence Framework (REF) |
| 1. **Kelly [29]** | Report | “However while the REF definition refers to ‘an effect on, change or benefit’ to the world outside academia, it is often difficult and frequently impossible to pin down evidence of ‘an actual ‘effect, change or benefit’ or prove that  a particular change  in policy, for example, was solely or  even partially attributable to a piece  of research. This is the ‘attribution problem’.” (p. 7) | UK Research Excellence Framework (REF) |
| 1. **Khazragui and Hudson [30]** | Journal article | “In the REF’s generic guidelines, impact is defined as ‘an effect on, change or benefit to the economy, society, culture, public policy or services, health, the environment or quality of life, beyond academia.’ Examples are given and include effects on, changes or benefits to the activity, attitude, awareness, behaviour, capacity, opportunity, performance, policy, practice, process or understanding of an audience, beneficiary, community, constituency, organisation or individuals. It is not bounded by location and can relate to local, national or international impact. It also emphasises that it includes the reduction or prevention of harm, risk, cost or other negative effects. But it specifically excludes impact on research or the advancement of academic knowledge within universities and in general impacts on students, teaching or other activities within the submitting university. (p. 5). | UK Research Excellence Framework (REF) |
| 1. **Marcella, Lockerbie and Cameron [11]** | Conference paper | “The REF defines impact as ‘an effect on, change or benefit to the economy, society, culture, public policy or services, health, the environment or quality of life, beyond academia’. Excluded from the definition is impact on research or academic knowledge within HE, as well as on students, teaching or other activities within Higher Education Institutes (HEIs).” (p. 2) | UK Research Excellence Framework (REF) |
| 1. **McKenna [31]** | Journal article | “A definition of Research Impact is research that has ’an effect on, change or benefit to the economy, society, culture, public policy or services, health, the environment or quality of life, beyond academia’. Research Impact includes an effect, change or benefit to: the activity, attitude, awareness, behaviour, capacity, opportunity, performance, policy, practice, process or understanding; an audience, beneficiary, community, constituency, organisation or individuals; any geographic location whether locally, regionally, nationally or internationally.” (p. 723-724) | Higher Education Funding Council for England (HEFCE) and UK Research Excellence Framework (REF) |
| 1. **Nightingale and Marshall [32]** | Journal article | “While citation data can provide information about the academic significance of an article, it does not necessarily provide evidence of research impact as defined for the purposes of REF 2014 research impact is characterised by ‘an effect on, change or benefit to the economy, society, culture, public policy or services, health, the environment or quality of life, beyond academia’.” (p. 439) | UK Research Excellence Framework (REF) |
| 1. **Ovseiko, Oancea and Buchan [33]** | Journal article | “For the purpose of the 2010 pilot exercise, HEFCE defined research impact as ‘any identifiable benefit to, or positive influence on, the economy, society, public policy or services, culture, the environment or quality of life,’ and provided HEIs with a ‘common menu’ of impact indicators in the following broad categories: Delivering highly skilled people; Creating new businesses, improving the performance of existing businesses, or commercialising new products or processes; Attracting R&D investment from global business; Improved patient care or health outcomes; Better informed public policy-making or improved public services; Progress towards sustainable development, including environmental sustainability; Cultural enrichment, including improved public engagement with science and research; Improved social welfare, social cohesion or national security; Other quality of life benefits.” (p. 3) | Higher Education Funding Council for England (HEFCE) |
| 1. **Pain, Kesby and Askins [34]** | Journal article | “In its second REF consultation, the Higher Education Funding Council for England suggests that for the first time a proportion of the government funding that it distributes for research will be allocated on the basis of the ‘demonstrable benefits [of research] to the wider economy and society’.” (p. 4-5) | Higher Education Funding Council for England (HEFCE) |
| 1. **Parker and van Teijlingen [35]** | Journal article | “The REF defines impact as ‘(a)ny identifiable benefit to or positive influence on the economy, society, public policy or services, culture, the environment and/or quality of life’. Impact includes, but is not limited to, an effect on, change or benefit to: the activity, attitude, awareness, behaviour, capacity, opportunity, performance, policy, practice, process or understanding; of an audience, beneficiary, community, constituency, organisation or individuals; and in any geographic location whether locally, regionally, nationally or internationally.” (p. 45) | UK Research Excellence Framework (REF) and the Higher Education Funding Council for England (HEFCE) |
| 1. **Penfield, Baker, Scoble and Wykes [36]** | Journal article | “The Oxford English Dictionary defines impact as a ‘Marked effect or influence’, this is clearly a very broad definition. In terms of research impact, organizations and stakeholders may be interested in specific aspects of impact, dependent on their focus. In this case, a specific definition may be required, for example, in the Research Excellence Framework (REF), ‘Assessment framework and guidance on submissions’, which defines ‘impact’ as,’ an effect on, change or benefit to the economy, society, culture, public policy or services, health, the environment or quality of life, beyond academia’.” (p. 21) | UK Research Excellence Framework (REF) |
| 1. **Reed [12]** | Book | “For example, the Higher Education Funding Council for England (HEFCE) defines impact as ‘an effect on, change or benefit to the economy, society, culture, public policy or services, health, the environment or quality of life, beyond academia’.” (p. 188) | Higher Education Funding Council for England (HEFCE) |
| 1. **Roberts, Madden and Corrall [37]** | Journal article | ‘The focus groups and the content analysis also highlight the distinction between communication or dissemination of research and formally recognized research impact (of the type defined and accepted by the U.K.REF).’ (p. 505) | UK Research Excellence Framework (REF) |
| 1. **Schnitzler, Davies, Ross and Harris [38]** | Journal article | ‘When the benefits from research are tangible, measurable and recognised outside of academia, research is said to have impact. This could be economic, environmental or cultural. Research impact can include changes to public policy, health care or quality of life. /-/ Research impact is defined as involving changes in practice, knowledge and understanding, attitudes and ideas resulting from research (Morton, 2015).’ (p. 16) | UK Research Excellence Framework (REF) and Morton, S., 2015a. Progressing research impact assessment: a ‘contributions’ approach. Res Eval. 24 (4), 405–419. |
| 1. **Sousa and Brennan [39]** | Book | ‘According to the HEFCE official website, the REF aims at the identification and reward of the impact that excellent research has had on society and the economy. /-/ Additionally, the reports made a number of recommendations regarding three themes: the definition of research impact – a broad definition, but excluding impact purely within academia -, the evidence of impact provided by institutions – construction of a narrative with case studies and indicators -, the assessment of impact by the REF panels – disciplinary specifics and robustness.’ (p. 73) | Higher Education Funding Council for England (HEFCE) |
| 1. **Wilkinson, Gallagher and Smith [40]** | Journal article | “The REF similarly encompasses ‘any social, economic or cultural impact or benefit beyond academia’ and also includes public engagement activities.” (p. 322) | UK Research Excellence Framework (REF) |

# **Table 2a. Research impacts as benefits, effects or changes (REF/HEFCE not cited)**

| *Author of Reference* | *Reference Type* | *Definition* | *Reference to Source of Definition* |
| --- | --- | --- | --- |
| 1. **Banzi, Moja, Pistotti, Facchini and Liberati [41]** | Journal article | "Broadly speaking the term ‘research impact’ refers to the contribution of research activities to achieve desired societal outcomes. (p. 1) /-/ In the context of this overview, the term ‘research impact’ refers to any type of output of research activities which can be considered a ‘positive return’ for the scientific community, health systems, patients, and the society in general.” (p. 2) | No reference (original) |
| 1. **Chandler [3]** | Book | “The Arts and Humanities Research Council (AHRC) has shown considerable interest in establishing impact within their remit and say, ‘By impact we mean the “influence” of research or its “effect on” an individual, a community, the development of policy, or the creation of a new product or service. It relates to the effects of research on our economic, social and cultural lives.’” (p. 2) | Arts and Humanities Research Council (AHRC) |
| 1. **Chandler [3]** | Book | ‘Impact in its simplest definition is about making a difference, so there is action or activity which leads to change, but that change needs to be seen within a context which may be global, local or even individual. Also, the nature of change needs to be considered, whether it is related to people, systems, environment, knowledge, understanding or policy. /-/ The nature of impact is identified as the influence, effect, demonstrable contribution, change or benefits that result from the research.’ (p. 2-3) | No reference (original) |
| 1. **Donovan [42]** | Journal article | “Research impact denotes the benefits or returns from research, which flow beyond the academic realm to ‘end users’ or research. These end-users are traditionally defined as industry, business, government, or more broadly, the taxpayer.” (p. 48) | No reference (original) |
| 1. **Drew, Pettibone, Finch, Giles and Jordan [43]** | Journal article | ‘For purposes of program and portfolio evaluation, we define impacts to be the benefits or changes resulting from scientific research, program activities or outputs.’ (p. 989) | National Institute  of Environmental Health Sciences (NIEHS) |
| 1. **Harland [44]** | Journal article | “The assessment exercise has caused substantial debate on ‘what is research impact?’ and philosophically and practically why it might be important and to whom. One definition of impact in The Oxford Dictionary is ‘a marked effect or influence’; in this paper we will examine who or what might be making the marked effect or influence and on whom or what it is being made.” (p. 486) | Oxford Dictionary |
| 1. **Primary Health Care Research and Information Service (PHCRIS), Beacham, Kalucy and McIntyre [45]** | Web report | ‘The term research impact describes the effects and outcomes, in terms of value and benefit, associated with the use of knowledge produced through research.’ (p. 3) | No reference (original) |
| 1. **Jones and Cleere [9]** | Web report | ‘The UCD Beyond Publications steering committee came to the decision that UCD’s focus on impact should be: The contribution of UCD’s research to the advancement of knowledge for the benefit of academia, the economy, society, culture, industry, public policy, health, the environment or quality of life.’ (p. 40) | No reference (original) |
| 1. **Kanefsky [46]** | Conference paper | “We have not found an entirely satisfactory definition of research ‘impact’ anywhere in the literature, whether from within the field of education or from other disciplines. Even the term itself is questioned by some, as to them it implies abrupt action not collaborative processes of influence. Perhaps the least unsatisfactory definition is that of the NERF subgroup report on impact (2000), which characterises impact as ‘the influence or effect that educational research has on its audiences’.” (p. 5) | NERF., The impact of educational research on policy and practice: sub group report. 2000. http://www.nerf-uk.org/subgroups/impact.html |
| 1. **Milat, Bauman and Redman [47]** | Journal article | “In the context of this review, research impact is defined as: … any type of output of research activities which can be considered a ‘positive return’ for the scientific community, health systems, patients, and the society in general.” (p. 2) | Banzi R, Moja L, Pistotti V, Facchini A, Liberati A. Conceptual frameworks and empirical approaches used to assess the impact of health research: an overview of reviews. Health Res Policy Syst. 2011;9:26. doi: 10.1186/1478-4505-9-26 |
| 1. **Reed [12]** | Book | “By ‘impact’, we are talking about beneficial changes that will happen in the real world (beyond the world of researchers) as a result of your research. This can include ‘negative impacts’ such as evidence that prevents the launch of a harmful product or law. Impacts may occur in the immediate or long-term future, and there can be challenges tracking and attributing impacts, which this book will help you explore.” (p. 171) | No reference (original) |
| 1. **Sarli, Dubinsky and Holmes [48]** | Journal article | “The Flinders University Primary Health Care Research and Information Service provided a definition of research impact that was adapted for this project: ‘’The term research impact describes the effects and outcomes, in terms of value and benefit, associated with the use of knowledge produced through research.’ Four stages of the research process were identified for the preliminary model: research output, knowledge transfer, clinical implementation, and community benefit. knowledge transfer, clinical implementation, and community benefit.” (p.18) | Primary Health Care Research and Information Service (PHCRIS) |
| 1. **Sanon, Evans-Agnew and Boutain [49]** | Journal article | ‘Impact in this article refers to how the research findings were used to promote change either at the individual level or in the systems, environment or policy realms.’ (p. 213) | No reference (original) |
| 1. **Solans-Domènech, Adam, Guillamón, Permanyer-Miralda, Pons and Escarrabill [50]** | Journal article | “In health sciences, the term ‘research impact’ refers to any type of output or outcome of research activities which can be considered a ‘positive return or payback’ for the scientific community, health systems, patients, and society in general.” (p. 2) | Grant J, Brutscher PB, Kirk SE, Butler L, Wooding S. Capturing research impacts a review of international practice. Cambridge: RAND Europe; 2010. |
| 1. **University of York [51]** | Website | “Research impact is, in general, the effect research has beyond academia. The York Research Impact Statement describes research impact as ‘…when the knowledge generated by our research contributes to, benefits and influences society, culture, our environment and the economy…/-/…translating research into real-world outcomes, benefitting health, prosperity and well-being of people and society.’” | No reference (original) |

# **Table 3. ARC/RQF definitions**

| *Author of Reference* | *Reference Type* | *Definition* | *Reference to Source of Definition* |
| --- | --- | --- | --- |
| 1. **The Academy of Technological Sciences and Engineering (ATSE) [52]** | Website report | “Broadly defined, research impact is the ‘demonstrable contribution that research makes to the economy, society, culture, national security, public policy or services, health, the environment, or quality of life, beyond contributions to academia’.” (p. 7) | Australian Research Council (ARC) |
| 1. **Australian Research Council (ARC) [53]** | Website | ‘Research impact is the demonstrable contribution that research makes to the economy, society, culture, national security, public policy or services, health, the environment, or quality of life, beyond contributions to academia.’ | No reference (original) |
| 1. **Bainbridge, Tsey, McCalman, Kinchin, Saunders, Watkin Lui, Cadet-James, Miller and Lawson [54]** | Journal article | “The Australian Research Council (ARC) defines research impact as ‘the demonstrable contribution that research makes to the economy, society, culture, national security, public policy or services, health, the environment, or quality of life, beyond contributions to academia’. It includes, but is not limited to, an effect on, change or benefit to: ‘the activity, attitude, awareness, behaviour, capacity, opportunity, performance, policy, practice, process or understanding of an audience, beneficiary, community, constituency, organisation or individuals in any geographic location whether locally, regionally, nationally or internationally’.” (p. 4) | Australian Research Council (ARC) |
| 1. **Birks and Mills [55]** | Book | “Research impact can be defined as 'the demonstrable contribution that research makes to the economy, society, culture, national security, public policy or services, health, the environment, or quality of life, beyond contributions to academia'.” (p. 158) | Australian Research Council (ARC) |
| 1. **Cleary, Sayers and Watson [56]** | Journal article | "However, the ARC definition of impact requires the researcher to make a demonstrable contribution ‘to the economy, society, culture, national security, public policy or services, health, the environment, or quality of life, beyond contributions to academia’. (p. 1) | Australian Research Council (ARC) |
| 1. **Commonwealth of Australia [57]** | Web report | ‘Research impact is defined as the social, economic, environmental and/or cultural benefit of research to end users in the wider community regionally, nationally, and/or internationally.’ (p. 21) | Australian Research Quality Framework (RQF) |
| 1. **Donovan [42]** | Journal article | “For example, the EAG had defined research impact as the ‘social, cultural, economic, and/or environmental outcomes for industry, government and/or other identified communities regionally within Australia, nationally and/or internationally’.” (p. 50) | Australian Research Quality Framework (RQF) |
| 1. **Donovan [42]** | Journal article | “The definition of impact is extended: ‘Impact refers to the extent to which research has led successfully to social, economic, environmental, and/or cultural benefits for the wider community, or an element of the community’, which allows inclusion of private value in addition to public value. In terms of defining research impact, there is an explicit request that research groups should include in their impact statements ‘identifiable and supportable impact-related indicators. This requires the impact statement to identify the beneficiaries of the research and the way in which they have benefited.’ The ‘push’ also dominates in the flavor of examples of impact given: ‘improved quality of products/services, cost-effectiveness, customer satisfaction, lives saved or productivity’; ‘policy impacts can also include changes to policies of corporations, councils, professional groups and non-government organizations’.” (p.57) | Australian Research Quality Framework (RQF) |
| 1. **Donovan [42]** | Journal article | “For RQF purposes, impact was originally concerned with social, economic, and environmental effects, reflecting a trend toward ‘triple bottom line’ accounting. The EAG’s consultation with the higher education sector led to introducing the ‘cultural’ as a fourth impact domain; and the resulting quadruple bottom line was unique in international impact assessment terms’.” (p.53) | Australian Research Quality Framework (RQF) |
| 1. **Doyle, Farley, Keppell, Cuthill and McDonald [23]** | Conference paper | “The ARC defines research impact as ‘the demonstrable contribution that research makes to the economy, society, culture, national security, public policy or services, health, the environment, or quality of life, beyond contributions to academia’.” (p. 638) | Australian Research Council (ARC) |
| 1. **Doyle and McDonald [24]** | Conference paper | "The ARC defines research impact as ‘the demonstrable contribution that research makes to the economy, society, culture, national security, public policy or services, health, the environment, or quality of life, beyond contributions to academia’." (p. 3) | Australian Research Council (ARC) |
| 1. **Duryea, Hochman and Parfitt [58]** | Journal article | “‘Research impact’ is defined within the RQF as the beneficial application of research to achieve social, economic, environmental and/or cultural outcomes. This is not to be confused with impact in the academic domain which is seen more as an indicator of the intrinsic quality of the research on scholarly or academic measures.” (p. 8) | Australian Research Quality Framework (RQF) |
| 1. **Gooch, Vasalou and Benton [59]** | Journal article | “Following from this position, the Australian Research Quality Framework (RQF), one of the first funding bodies to establish a definition of impact, focuses on the economic, social, environmental and cultural benefits of research. The so called ‘quadruple bottom line’ approach has come to dominate, with the four aspects of social, economic, cultural and environmental benefits being seen to cover the range of contributions which may be regarded as constituting impact’.” (p. 2) | Australian Research Quality Framework (RQF) |
| 1. **Harris, Thieberger and Barwick [60]** | Book | “The ARC’s definition of impact is ‘the demonstrable contribution that research makes to the economy, society, culture, national security, public policy or services, health, the environment, or quality of life, beyond contributions to academia’.” (p. 32) | Australian Research Council (ARC) |
| 1. **O'Brien [61]** | Conference paper | “Within Australia the Research Quality Framework (RQF) was introduced in 2005 to follow in the footsteps of other nation’s research quality frameworks. The RQF differed from existing international research assessment exercises in that it sought to measure ‘research impact’. ‘Research impact’ was defined as “the beneficial application of research to achieve social, economic, environmental and/or cultural outcomes.” Measures of impact included analyses of patents, cost-benefit assessments, social returns and citations.” (p. 147) | Australian Research Quality Framework (RQF) |
| 1. **Onslow [62]** | Journal article | “Research Impact is the social, economic, environmental and/or cultural benefit of research to end-users outside the peer academic community. /-/ And according to the Australian Government, your research has made an impact if it produces ‘identifiable social, economic, environmental and/or cultural benefit for the wider community’.” (p. 120) | Australian Research Quality Framework (RQF) |
| 1. **Rekhi and Lane [63]** | Journal article | “For example, in 2008, Australia, one of the world’s leaders in the percentage of GDP it devotes to scientific research, replaced its quantitative metrics paradigm with a more qualitative ‘Research Quality Framework,’ which includes panel assessments of ‘impact in the form of the social, economic, environmental and cultural returns of research beyond the academic peer community’.” (p. 24) | Australian Research Quality Framework (RQF) |

# **Table 4. Bibliometric definitions**

| *Author of Reference* | *Reference Type* | *Definition* | *Reference to Source of Definition* |
| --- | --- | --- | --- |
| 1. **Association of Commonwealth Universities (ACU) [18]** | Website | “The LSE’s maximising impact handbook, though, defines ‘a research impact’ as ‘an occasion of influence’ rather than what might happen as a result of that influence.” | London School of Economics and Political Science Public Policy Group (PPG) |
| 1. **Bainbridge, Tsey, McCalman, Kinchin, Saunders, Watkin Lui, Cadet-James, Miller and Lawson [54]** | Journal article | “A group from the United Kingdom who have been particularly active in the field of research impact, the London School of Economics (LSE) Public Policy Group assert that it is ‘an occasion of influence and hence it is not the same thing as a change in outputs or activities as a result of that influence, still less a change in social outcomes’. They suggest two broad areas of impact from which benefits are derived: 1) Academic impacts – influences upon actors in academia or universities e.g., as measured by citations in other academic works; and 2) External impacts – influences on actors outside higher education, in business, government or civil society e.g., as measured by references in government documents.” (p. 4) | London School of Economics and Political Science Public Policy Group (PPG) |
| 1. **Bornmann [64]** | Journal article | “The problem actually begins with defining the ‘societal impact of research’. A series of different concepts has been introduced: ‘third-stream activities’, ‘societal benefits’ or ‘societal quality’, ‘usefulness’, ‘public values’, ‘knowledge transfer’ and ‘societal relevance’. Yet, each of these concepts is ultimately concerned with measuring the social, cultural, environmental and economic returns from publicly funded research, be they products or ideas.” (p. 673) | No reference (original) |
| 1. **Brewer [2]** | Journal article | “The ESRC’s website goes on to say: ‘research impact embraces all the diverse ways that research-related skills benefit individuals, organisations and nations. These include: fostering global economic competitiveness of the United Kingdom increasing the effectiveness of public services and policy; enhancing quality of life, health and creative output. A key aspect of this definition of research impact is that impact must be demonstrable. It is not enough just to focus on activities and outputs that promote research impact, such as staging a conference or publishing a report. You must be able to provide evidence of research impact, for example, that it has been taken up and used by policy makers, and practitioners, has led to improvements in services and business’.” (p. 88) | Economic and Social Research Council (ESRC) |
| 1. **Buykx, Humphreys, Wakerman, Perkins, Lyle, McGrail and Kinsman [65]** | Journal article | “For our purposes, we acknowledge the distinction between KT, which refers to activities or processes that facilitate the use of research knowledge, and RI [research impact], which refers to outcomes or measures of the use of research knowledge, that is ‘auditable occasions of influence’.” (p. 51) | No reference (original) |
| 1. **Cohen, Schroeder, Newson, King, Rychetnik, Milat, Bauman, Redman and Chapman [66]** | Journal article | ‘Policy or practice impacts were defined as demonstrable changes, or benefits to products, processes, policies, and or practices, that occur after a research project has concluded. These impacts are concrete, measurable changes in policy or practice such as a new government policy, a change in organizational or clinical practice, a health education campaign or related new funding that can be attributed to the research intervention in question. Impacts at this level could also include stopping or changing existing interventions following demonstration of intervention ineffectiveness. Policy or practice impacts can be widespread or localized, and may benefit specific or general populations.’ (p. 2) | No reference (original) |
| 1. **Drummond [67]** | Journal article | “The London School of Economics defines research impact as a ‘recorded or otherwise auditable occasion of influence from academic research on another actor or organization’.” (p. 310) | London School of Economics and Political Science Public Policy Group (PPG) |
| 1. **Harland [44]** | Journal article | ‘In Finland the quality of research, research activity, impact of research, activity in educating young scientists and activity in the scientific community are identified as appropriate criteria for assessing quality of research; within this assessment, research impact is defined in terms of citations by other researchers (in journal articles, books, published conference proceedings, and PhD dissertations), invited and plenary presentations in international conferences, and the number of foreign co-authors in journal articles.’ (p. 488) | Korhonen P, Tainio R, Wallenius J. Value efficiency analysis of academic research. Eur J Oper Res. 2001 Apr 1;130(1):121-32. |
| 1. **Hannemann-Weber, Kessel and Schultz [68]** | Journal article | ‘To investigate a CoEs research performance, we distinguish between the output and impact. According to Moed et al., research output is the extent to which a body of scientific results is created. The impact of publications is defined as the amount of influence the research output has on other person’s activities. Although numerous publications are produced, only a few are accepted by the scientific community and subsequently included in further research and applications. Research impact is not exclusively an indicator of quality; it also reflects the reputation and related visibility of a CoE within the community and a CoEs efforts to transfer results. Therefore, we examined research output in terms of quantity of publications and research impact by number of citations. Bibliometric data are seen as an objective quantitative indicator for scientific performance.’ (p. 141-142) | Moed HF, Burger JM, Frankfort JG, Van Raan AFJ. The use of bibliometric data for the measurement of university research performance.  Res Policy 1985;14(3):131-49. |
| 1. **Kelly [29]** | Report | “The LSE Handbook on Maximising the Impacts of Your Research – which is one of the most recent (and helpful) documents on research impact around at the moment  – tries to deal with  this by narrowing the definition of ‘impact’ from an actual change or ‘benefit’  to an ‘occasion of influence’.  The LSE handbook definition of a non‐academic research impact is: ‘a recorded or otherwise auditable occasion of influence from academic research on another actor or organisation‘.” (p. 7) | London School of Economics and Political Science Public Policy Group (PPG) |
| 1. **LSE Public Policy Group (PPG) [69]** | Web report | ‘We define a research impact as a recorded or otherwise auditable occasion of influence from academic research on another actor or organization. Impact is usually demonstrated by pointing to a record of the active consultation, consideration, citation, discussion, referencing or use of a piece of research.’ (p. 11) | No reference (original). |
| 1. **Moed, Burger, Frankfort and Van Raan [70]** | Journal article | ‘In this paper we are concerned with two important aspects of research performance: output and impact. Output refers to the extent to which the research creates a body of scientific results. Impact is defined as the actual influence of the research output on surrounding research activities.’ (p. 132) | No reference (original) |
| 1. **National Health and Medical Research Council (NHMRC) [71]** | Website | “Measuring citations in publications is just one measure of the ‘impact’ of research; it’s simply the impact on the published ideas or methods on other scientists, as acknowledged in their own publications. It’s much harder to measure the other impacts of research; those that contribute to new commercial opportunities, better products and processes to treat patients, better policies to improve prevention strategies, publications that influence policy makers to make improvements in management and administration of our system, and even how being a researcher and a teacher or clinician improves your work.” | No reference (original) |
| 1. **Qin [72]** | Conference paper | ‘On a macro-level of research impact, three factors will determine the overall impact of research: the geographical and disciplinary extent to which research output has been diffused, the adoption where the overall impact I is defined as the product of the extent (E) of knowledge diffusion, in which citation data can be utilized to analyze the rate and scope of knowledge diffusion, the rate of adoption (A) as represented by the proportion of intellectual property that has been licensed or purchased among all produced, and the benefits (B) to society in both quantitative and qualitative terms.’ (p. 2) | No reference (original) |
| 1. **Tonta, Ünal and Al [73]** | Conference paper | “The term ‘research impact’ in this study is defined as the number of times that each article is cited in the literature.” (p. 2) | No reference (original) |

# **Table 5. Use-based definitions**

| *Author of Reference* | *Reference Type* | *Definition* | *Reference to Source of Definition* |
| --- | --- | --- | --- |
| 1. **Cox, Cozzens, van Ark, McCauley and Borbey [74]** | Web report | “Before analysing the social and cultural impacts of research, it is first necessary to define what we mean by ‘impact’. The impact(s) of research occur when others besides the research group itself notice the results, refer to these results, use the results or even commission further research. Outcomes of research can eventually lead to societal changes, generally called societal impact. Societal impact can be further differentiated into social (public sector), economic (private sector) and cultural impacts; however these impacts often overlap.” (p. 26) | No reference (original) |
| 1. **Jones and Cleere [9]** | Web report | ‘ESF classify various forms of impact as outlined alphabetically below: Cultural impact: contribution to understanding of ideas and reality, values and beliefs. Economic impact: contribution to the sale price of products, a firm’s costs and revenues (micro level), and economic returns either through economic growth or productivity growth (macro level). Environmental impact: contribution to the management of the environment, for example, natural resources, environmental pollution, climate and meteorology. Health impact: contribution to public health, life expectancy, prevention of illnesses and quality of life. Political impact: contribution to how policy makers act and how policies are constructed and to political stability. Scientific impact: contribution to the subsequent progress of knowledge, the formation of disciplines, training and capacity building. Social impact: contribution to community welfare, quality of life, behaviour, practices and activities of people and groups. Technological impact: contribution to the creation of product, process and service innovations. Training impacts: contribution to curricula, pedagogical tools, qualifications.’ (p. 19) | European Science Foundation (ESF) |
| 1. **Hargreaves [75]** | Web report | ‘Research impact is defined as the application, use and influence of research across  various categories.’ (p. 7) | No reference (original) |
| 1. **Meagher, Lyall and Nutley [76]** | Journal article | “Although other definitions exist for the main types of research use and impacts, in this study we use the following definitions: ‘Instrumental use or impact’ refers to the direct impact of research on policy and practice decisions where a specific piece of research is used in making a specific decision or in defining the solution to a specific problem. ‘Conceptual use or impact’ is a more wide-ranging definition of research use, comprising the complex and often indirect ways in which research can have an impact on the knowledge, understanding and attitudes of policy-makers and practitioners.” (p. 165). | Nutley SM, Walter I, Davies HT: Using evidence: how research can inform public services. Bristol, UK: Policy Press; 2007. |
| 1. **Morton [77]** | Journal article | ‘In order to define research impact, the spectrum of impact from conceptual to instrumental set out by Nutley et al (2007) was used as a starting point. Changes in awareness, knowledge and understanding, ideas, attitudes and perceptions, and policy and practice can be considered impacts across this scale.’ (p. 36) | Nutley SM, Walter I, Davies HT: Using evidence: how research can inform public services. Bristol, UK: Policy Press; 2007. |
| 1. **Nutley, Walter and Davies [78]** | Book | “Broadly, instrumental use refers to the direct impact of research on policy and practice decisions. It identifies the influence of a specific piece of research in making a specific decision or in defining the solution to a specific problem, and represents a widely held view of what research use means. Conceptual use is a much more wide-ranging definition of research use, comprising the complex and often indirect ways in which research can have an impact on the knowledge, understanding and attitudes of policy makers and practitioners. It happens where research changes ways of thinking, alerting policy makers and practitioners to an issue or playing a more general ‘consciousness-raising’ role. Such uses of research may be less demonstrable but are no less important than more instrumental forms of use.” (p. 36) | No reference (original). |
| 1. **Sumner, Crichton, Theobald, Zulu and Parkhurst [79]** | Journal article | ‘In terms of the research impact on policy, Davies and colleagues explain: Non-academic research impact is about identifying the influences of research findings on policy, managerial and professional practices, social behaviour or public discourse. Such impact may be instrumental, influencing changes in policy, practices and behaviour, or conceptual, changing people’s knowledge, understanding and attitudes towards social issues…research can contribute not just to decisional choices, but also to the formation of values, the creation of new understandings and possibilities, and to the quality of public and professional discourse and debate.’ (p. 3) | Davies HT, Nutley SM, Walter I. In: Assessing the impact of social science research: conceptual, methodological and practical issues. A background discussion paper for ESRC Symposium on Assessing Non-Academic Impact of Research. St. Andrews: Research Unit for Research Utilisation; 2005. |
| 1. **Walter, Davies and Nutley [80]** | Journal article | ‘Within this review, research impact was defined along a continuum, ranging from raising awareness of findings, through increasing knowledge and understanding, to actual changes in decision-making or decisions. These can be thought of as conceptual uses at one end of the continuum and instrumental uses at the other.’ (p. S2: 59) | No reference (original) |
| 1. **Wilkinson, Gallagher and Smith [40]** | Journal article | ‘The Economic and Social Research Council adopts a broad understanding of impact, encompassing: economic benefits; effects on the environment, public health and quality of life; impacts on policy and practice in governmental, private and third sector organisations; knowledge exchange impacts, such as relationships that might facilitate future knowledge exchange.’ (p. 322) | Armstrong F, Alsop A. Debate: co-production can contribute to research impact in the social sciences. Public Money & Management. 2010 Jul 1;30(4):208-10. |

# **Table 6. Original definitions**

| *Author of Reference* | *Reference Type* | *Definition* | *Reference to Source of Definition* |
| --- | --- | --- | --- |
| 1. **Canadian Institute of Health Research (CIHR) [81]** | Web report | ‘Impacts: In the context of evaluating health research, the overall results of all the effects of a body of research have on society. Impact includes outputs and outcomes, and may also include additional contributions to the health sector or to society. Impact includes effects that may not have been part of the research objectives, such as contributions to a knowledge based society or to economic growth.’ (p. 22) | No reference (original) |
| 1. **Drew, Pettibone, Finch, Giles and Jordan [43]** | Journal article | ‘Impacts, often called outcomes, are the effects of the research on the research field or within society.’ (p. 989) | The Kellogg Foundation |
| 1. **Eisenberg [82]** | Journal article | ‘To address this need to demonstrate the impact of research on people’s health, we can use a model that shows different levels of the impact of research. This model was developed by AHRQ staff and consultants who conceived of a pyramid of outcomes that included four different levels of impact (Figure 1), beginning (at the pyramid’s bottom) with impact on knowledge and further research (level one) and ascending (at the pyramid’s peak) to impact on health outcomes (level four). In between are impact on policies (level two) and impact on clinical practice (level three). The first level of impact represents research that contributes to the health care knowledge base, leads to future research, or both. Level one also includes tools and methods for research, instruments and techniques to assist clinical decision making, and studies that identify areas in with scientific knowledge is absent but needed. (p. xii) /-/ The second level of impact is research that results in the creation of a policy or program (for example, by professional organizations, health plans, hospitals, legislative bodies, regulators, or accrediting organizations). /-/ At level three, research impact is defined as research that results in a change in what clinicians or patients do, or changes in a pattern of care. /-/ Examples of level four, that is, impact on actual health outcomes, include reduction of treatment costs and waiting room time for HIV-positive patients through use of the CHESS system and the following example on stroke prevention…’ (p. xiii) | No reference (original) |
| 1. **Gooch, Vasalou and Benton [59]** | Journal article | ‘The current approach [to defining research impact] instead focuses on delineating types of impact. As this is the approach used by funding bodies who evaluate research impact, there has been little incentive to consider other approaches although Abreu et al. (2009) make an attempt to define the types of impactful activities, namely people-based, community-based, commercialisation and problem-solving.’ (p. 2) | Abreu M, Grinevich V, Hughes A, Kitson M. Knowledge exchange between academics and the business, public and third sectors. UK-Innovation Research Centre; 2009. http://eprints.soton.ac.uk/357117/1/AcademicSurveyReport.pdf |
| 1. **Thonon, Boulkedid, Delory, Rousseau, Saghatchian, van Harten, O'Neill and Alberti [83]** | Journal article | “We use the definition of impact proposed by the Canadian Institute of Health Research: ‘In the context of evaluating health research, the overall results of all the effects of a body of research have on society. Impact includes outputs and outcomes, and may also include additional contributions to the health sector or to society. Impact includes effects that may not have been part of the research objectives, such as contributions to a knowledge based society or to economic growth.’ In this article we make a distinction between scientific impact and health service impact.” (p. 2) | Canadian Institute of Health Research (CIHR) |

# **References**

1. Bannister J, Hardill I: **Knowledge mobilisation and the social sciences: dancing with new partners in an age of austerity.** *Contemporary Social Science* 2013, **8:**167-175. doi:10.1080/21582041.2013.770910

2. Brewer JD: *The public value of the social sciences: An interpretive essay.* London, UK: Bloomsbury; 2013.

3. Chandler C: **What is the meaning of impact in relation to research and why does it matter? A view from inside academia.** In *Achieving impact in research.* Edited by Denicolo P. Los Angeles, LA: Sage; 2013

4. Clegg B, Lightfoot H, Scully J: **Identifying capacity building cluster impact in the West Midlands.** 2013.

5. Economic and Social Research Council: **What is impact?** [http://www.esrc.ac.uk/research/evaluation-and-impact/what-is-impact/]. Accessed 8 November 2015.

6. Halse C, Mowbray S: **The impact of the doctorate.** *Stud High Educ* 2011, **36:**513-525. doi:10.1080/03075079.2011.594590

7. Holt N, Lewis R: *Crown House AQA Psychology AS level and year 1.* Carmarthen, Wales, UK: Crown House Publishing; 2015.

8. Jansson K, Karvonen I, Kettunen O, Ollus M: **Forecasting impact of technology developed in R&D projects: the FITMAN approach.** [http://eprints.soton.ac.uk/379716/1/379716.pdf]. Accessed 20 February 2016.

9. Jones A, Cleere L: **Furthering the research impact of UCD: Report of the Beyond Publications Committee.** [http://irserver.ucd.ie/bitstream/handle/10197/7292/Furthering_Impact_May_2014.pdf?sequence=1]. Accessed 20 February 2016.

10. Kraatz JA, Hampson KD, Parker RL, Roos G: **What next? Future directions for R&D investment.** In *R&D investment and impact in the global construction industry.* Edited by Hampson KD, Kraatz JA, Sanchez AX. Abingdon, UK: Routledge; 2014: 284-309

11. Marcella R, Lockerbie H, Cameron R: **The challenge of demonstrating the impact of research beyond traditional mechanisms.** In *14th European Conference on Research Methodology for Business and Management Studies (ECRM 2015); University of Malta, Valletta, Malta (11-12 June 2015)*. Edited by Bezzina F, Cassar V. Academic Conferencees and Publishing International Limited; 2015: 259-266.

12. Reed MS: *The research impact handbook.* St Johns Well, Scotland: Fast Track Impact; 2016.

13. Research Councils UK (RCUK): **Excellence with impact.** [http://www.rcuk.ac.uk/innovation/impact]. Accessed 1 October 2014.

14. Shaw I: *Social work science.* New York: Columbia University Press; 2016.

15. Shaw I, Holland S: *Doing qualitative research in social work.* London, UK: Sage; 2014.

16. SOAS University of London: **Impact Acceleration Fund: Definition of impact.** [https://www.soas.ac.uk/researchoffice/iaf/]. Accessed 20 February 2016.

17. Weitkamp E: **Between ambition and evidence.** *Sci Commun* 2015, **14:**1-5.

18. Association of Commonwealth Universities (ACU): **Defining, understanding and measuring impact.** [https://www.acu.ac.uk/membership/acu-insights/acu-insights-2/defining-understanding-and-measuring-impact]. Accessed 3 June 2016.

19. Bernal I: **Open access and the changing landscape of research impact indicators: New roles for repositories.** *Publications* 2013, **1:**56-77. doi:10.3390/publications1020056

20. Harris R: **The impact of research on development policy and practice: This much we know.** In *Impact of information society research in the Global South.* Edited by Chib A, May J, Barrantes R. Singapore: SpringerOpen; 2015: 21-44

21. Colley H: **What (a) to do about ‘impact’: a Bourdieusian critique.** *Brit Educ Res J* 2014, **40:**660-681. doi:10.1002/berj.3112

22. Détourbe M-A: **From public funding to public investment in research: a study of research funding policies and their impact through two research assessment campaigns in the United Kingdom.** [http://lisa.revues.org/8903#quotation]. Accessed 25 April 2016.

23. Doyle J, Farley H, Keppell M, Cuthill M, McDonald L: **Three good reasons to understand the research impact of a technology-enabled initiative.** In *Rhetoric and reality: Critical perspectives on educational technology Proceedings ascilite Dunedin 2014.* Edited by Hegarty B, McDonald J, Loke S-K; 2014: 638-642

24. Doyle J, McDonald L: **Making an impact: politics and persuasions in 21st century higher education.** [http://eprints.usq.edu.au/28371/1/DoyleMcDonald.pdf]. Accessed 14 February 2016.

25. Greenhalgh T, Fahy N: **Research impact in the community-based health sciences: an analysis of 162 case studies from the 2014 UK Research Excellence Framework.** *BMC Med* 2015, **13:**232-246. doi:10.1186/s12916-015-0467-4

26. Hartwell H, van Teijlingen E, Parker J: **Nutrition; effects of the Research Excellence Framework (REF).** *Nutr Food Sci* 2013, **43:**74-77. doi:10.1108/00346651311295941

27. Higher Education Funding Council from England (HEFCE): **REF impact.** [http://www.hefce.ac.uk/rsrch/REFimpact]. Accessed 27 February 2016.

28. Holt GD, Goulding JS, Akintoye A: **Enablers, challenges and relationships between research impact and theory generation.** *Engineering, Construction and Architectural Management* 2016, **23:**20-39. doi:10.1108/ECAM-12-2014-0161

29. Kelly U: **The ‘Impact Analysis System’: Project report and guide to the underpinning conceptual framework.** [http://www.viewforthconsulting.co.uk/sitebuildercontent/sitebuilderfiles/finalrep.pdf]. Accessed 10 February 2016.

30. Khazragui H, Hudson J: **Measuring the benefits of university research: impact and the REF in the UK.** *Res Eval* 2015, **24:**51-62. doi:10.1093/reseval/rvu028

31. McKenna HP: **Perspectives: Patient and public involvement and research impact: a reciprocal relationship.** *J Res Nurs* 2015, **20:**723-728. doi:10.1177/1744987115619803

32. Nightingale JM, Marshall G: **Reprint of "Citation analysis as a measure of article quality, journal influence and individual researcher performance".** *Nurse Educ Pract* 2013, **13:**429-436. doi:10.1038/465860a

33. Ovseiko PV, Oancea A, Buchan AM: **Assessing research impact in academic clinical medicine: a study using Research Excellence Framework pilot impact indicators.** *BMC Health Serv Res* 2012, **12:478:**1-23. doi:10.1186/1472-6963-12-478

34. Pain R, Kesby M, Askins K: **Geographies of impact: power, participation and potential.** *Area* 2011, **43:**183-188. doi:10.1111/j.1475-4762.2010.00978.x

35. Parker J, van Teijlingen E: **The Research Excellence Framework (REF): Assessing the impact of social work research on society.** *Practice* 2012, **24:**41-52. doi:10.1080/09503153.2011.647682

36. Penfield T, Baker MJ, Scoble R, Wykes MC: **Assessment, evaluations, and definitions of research impact: A review.** *Res Eval* 2014, **23:**21-32. doi:10.1093/reseval/rvt021

37. Roberts A, Madden AD, Corrall S: **Putting research into practice: an exploration of Sheffield iSchool approaches to connecting research with practice.** *Libr Trends* 2013, **61:**479-512. doi:10.1353/lib.2013.0006

38. Schnitzler K, Davies N, Ross F, Harris R: **Using Twitter™ to drive research impact: A discussion of strategies, opportunities and challenges.** *Int J Nurs Stud* 2016, **59:**15-26. doi:10.1016/j.ijnurstu.2016.02.004

39. Sousa SB, Brennan JL: **The UK Research Excellence Framework and the transformation of research production.** In *Reforming higher education: Public policy design and implementation.* Edited by Musselin C, Teixeira PN. Dordrecht, Netherlands: Springer; 2014: 65-82

40. Wilkinson H, Gallagher M, Smith M: **A collaborative approach to defining the usefulness of impact: lessons from a knowledge exchange project involving academics and social work practitioners.** *Evid Policy* 2012, **8:**311-327. doi:10.1332/174426412X654040

41. Banzi R, Moja L, Pistotti V, Facchini A, Liberati A: **Conceptual frameworks and empirical approaches used to assess the impact of health research: An overview of reviews.** *Health Res Policy Syst* 2011, **9:26:**1-10. doi:10.1186/1478-4505-9-26

42. Donovan C: **The Australian Research Quality Framework: A live experiment in capturing the social, economic, environmental, and cultural returns of publicly funded research.** *New Directions for Evaluation* 2008, **2008:**47-60. doi:10.1002/ev.260

43. Drew CH, Pettibone KG, Finch FO, Giles D, Jordan P: **Automated research impact assessment: a new bibliometrics approach.** *Scientometrics* 2016, **106:**987-1005. doi:10.1007/s11192-015-1828-7

44. Harland CM: **Supply chain management research impact: an evidence‐based perspective.** *Supply Chain Management: An International Journal* 2013, **18:**483-496. doi:10.1108/SCM-03-2013-0108

45. Primary Health Care Research and Information Service (PHCRIS), Beacham B, Kalucy L, McIntyre E: *FOCUS on...Understanding & measuring research impact.* Adelaide, Australia: Primary Health Care Research and Information Service (PHCRIS); 2005.

46. Kanefsky J: **Research impact and the ESRC Teaching and Learning Research Programme.** In *British Educational Research Association Annual Conference*. University of Leeds, Leeds, UK (September 2001); 2001.

47. Milat AJ, Bauman AE, Redman S: **A narrative review of research impact assessment models and methods.** *Health Res Policy Syst* 2015, **13:**1-7. doi:10.1186/s12961-015-0003-1

48. Sarli CC, Dubinsky EK, Holmes KL: **Beyond citation analysis: a model for assessment of research impact.** *J Med Lib Assoc* 2010, **98:**17-23. doi:10.3163/1536-5050.98.1.008

49. Sanon M-A, Evans-Agnew RA, Boutain DM: **An exploration of social justice intent in photovoice research studies from 2008 to 2013.** *Nurs Inq* 2014, **21:**212-226. doi:10.1111/nin.12064

50. Solans-Domènech M, Adam P, Guillamón I, Permanyer-Miralda G, Pons JM, Escarrabill J: **Impact of clinical and health services research projects on decision-making: a qualitative study.** *Health Res Policy Syst* 2013, **11:15:**1-11. doi:10.1186/1478-4505-11-15

51. University of York: **What is research impact?** [https://www.york.ac.uk/staff/research/research-impact/impact-definition]. Accessed 14 October 2015.

52. The Academy of Technological Sciences and Engineering (ATSE): **Research engagement for Australia: Measuring research engagement between universities and end users.** Melbourne, Australia: Australian Academy of Technological Sciences and Engineering (ATSE); 2015.

53. Australian Research Council (ARC): **Research impact principles and framework.** [http://www.arc.gov.au/research-impact-principles-and-framework#Definition]. Accessed 14 October 2015.

54. Bainbridge R, Tsey K, McCalman J, Kinchin I, Saunders V, Watkin Lui F, Cadet-James Y, Miller A, Lawson K: **No one's discussing the elephant in the room: contemplating questions of research impact and benefit in Aboriginal and Torres Strait Islander Australian health research.** *BMC Public Health* 2015, **15: 696:**1-10. doi:10.1186/s12889-015-2052-3

55. Birks M, Mills J: *Grounded theory: A practical guide.* London, UK: Sage; 2015.

56. Cleary M, Sayers J, Watson R: **Writing research impact statements.** *Nurse Author & Editor* 2016, **26:**4.

57. Commonwealth of Australia: **Research Quality Framework: Assessing the quality and impact of research in Australia. The recommended RQF.** [https://research.vu.edu.au/ordsite/management/Recommended_RQF_Dec2006.pdf]. Accessed 2 January 2016.

58. Duryea M, Hochman M, Parfitt A: **Measuring the impact of research.** *Research Global* 2007, **1:**8-9.

59. Gooch D, Vasalou A, Benton L: **Impact in interdisciplinary and cross-sector research: Opportunities and challenges.** *J Assoc Inf Sci Technol* 2015, **March:**1-18. doi:10.1002/asi.23658

60. Harris A, Thieberger N, Barwick L: *Research, records and responsibility: Ten years of PARADISEC.* Sydney, Australia: Sydney University Press; 2015.

61. O'Brien L: **The changing scholarly information landscape: Reinventing information services to increase research impact.** In *'Publishing in the networked world: transforming the nature of communication 14th International Conference on Electronic Publishing'*; *16 - 18 June 2010; Helsinki, Finland (16-18 June 2010)*. Edited by Hedlund T, Tonta Y. 2010: 142-166.

62. Onslow M: **Eternity and clinical translation of speech-language pathology research.** *Int J Speech Lang Pathol* 2008, **10:**118-126. doi:10.1080/17549500801891632

63. Rekhi R, Lane N: **Qualitative metrics in science policy: What can't be counted, counts.** *Issues Sci Technol* 2012, **29:**21-24.

64. Bornmann L: **Measuring the societal impact of research.** *Sci Soc* 2012, **13:**673-676. doi:10.1038/embor.2012.99

65. Buykx P, Humphreys J, Wakerman J, Perkins D, Lyle D, McGrail M, Kinsman L: **'Making evidence count': a framework to monitor the impact of health services research.** *Aust J Rural Health* 2012, **20:**51-58. doi:10.1111/j.1440-1584.2012.01256.x

66. Cohen G, Schroeder J, Newson R, King L, Rychetnik L, Milat AJ, Bauman AE, Redman S, Chapman S: **Does health intervention research have real world policy and practice impacts: Testing a new impact assessment tool.** *Health Res Policy Syst* 2015, **13:3:**1-12. doi:0.1186/1478-4505-13-3

67. Drummond R: **RIMS revisited: The evolution of the research impact measurement service at UNSW library.** *Australian Academic & Research Libraries* 2014, **45:**309-322. doi:10.1080/00048623.2014.945065

68. Hannemann-Weber H, Kessel M, Schultz C: **Research performance of centers of expertise for rare diseases – the influence of network integration, internal resource access and operational experience.** *Health Policy* 2012, **105:**138-145. doi:10.1016/j.healthpol.2012.02.008

69. LSE Public Policy Group (PPG): **Maximising the impacts of your research: A handbook for social scientists.** [http://www.lse.ac.uk/government/research/resgroups/LSEPublicPolicy/Docs/LSE_Impact_Handbook_April_2011.pdf]. Accessed 27 June 2016.

70. Moed HF, Burger WJM, Frankfort JG, Van Raan AFJ: **The use of bibliometric data for the measurement of university research performance.** *Res Policy* 1985, **14:**131-149. doi:10.1016/0048-7333(85)90012-5

71. National Health and Medical Research Council (NHMRC): **Measuring the impact of research – not just a simple list of publications.** [https://www.nhmrc.gov.au/media/newsletters/ceo/2014/measuring-impact-research-not-just-simple-list-publications]. Accessed 26 November 2016.

72. Qin J: **Empirically assessing impact of scholarly research.** In *iConference 2010; Urbana-Champaign, IL, USA (3–6 February, 2010)*. 2010

73. Tonta Y, Ünal Y, Al U: **The research impact of open access journal articles.** In *11th International Conference on Electronic Publishing; Vienna, Austria (13-15 June 2007)*. 2007

74. Cox D, Cozzens S, van Ark G, McCauley L, Borbey P: *Evaluation of impacts of medical research.* Bromma, Sweden: Swedish Research Council; 2010.

75. Hargreaves J: *Assessing the impact of research: a case study of the LSAY Research Innovation and Expansion Fund.* Adelaide, Australia: National Centre for Vocational Education Research; 2012.

76. Meagher L, Lyall C, Nutley S: **Flows of knowledge, expertise and influence: a method for assessing policy and practice impacts from social science research.** *Res Eval* 2008, **17:**163-173. doi:10.3152/095820208X331720

77. Morton S: **Creating research impact: the roles of research users in interactive research mobilisation.** *Evid Policy* 2015, **11:**35-55. doi:10.1332/174426514X13976529631798

78. Nutley SM, Walter I, Davies HT: *Using evidence: how research can inform public services.* Bristol, UK: Policy Press; 2007.

79. Sumner A, Crichton J, Theobald S, Zulu E, Parkhurst J: **What shapes research impact on policy? Understanding research uptake in sexual and reproductive health policy processes in resource poor contexts.** *Health Res Policy Syst* 2011, **9 (Suppl 1): S3:**1-10. doi:10.1186/1478-4505-9-S1-S3

80. Walter I, Davies H, Nutley S: **Increasing research impact through partnerships: evidence from outside health care.** *J Health Serv Res Policy* 2003, **8:**58-61. doi:10.1258/135581903322405180

81. Canadian Institute of Health Research (CIHR): *Developing a CIHR framework to measure the impact of health research: A framework for measuring the impact of health research.* Ottawa, Canada: Canadian Institute of Health Research; 2005.

82. Eisenberg JM: **Putting research to work: reporting and enhancing the impact of health services research.** *Health Serv Res* 2001, **36:**x-xvii.

83. Thonon F, Boulkedid R, Delory T, Rousseau S, Saghatchian M, van Harten W, O'Neill C, Alberti C: **Measuring the outcome of biomedical research: a systematic literature review.** *Plos One* 2015, **10:**1-14. doi:10.1371/journal.pone.0122239
